# Circulation of HRSV in Belgium: From Multiple Genotype Circulation to Prolonged Circulation of Predominant Genotypes

**DOI:** 10.1371/journal.pone.0060416

**Published:** 2013-04-05

**Authors:** Lieselot Houspie, Philippe Lemey, Els Keyaerts, Eva Reijmen, Valentijn Vergote, Anne Vankeerberghen, Freya Vaeyens, Hans De Beenhouwer, Marc Van Ranst

**Affiliations:** 1 Laboratory of Clinical Virology, Rega Institute for Medical Research, University of Leuven, Leuven, Belgium; 2 Laboratory of Clinical and Epidemiological Virology, Rega Institute for Medical Research, University of Leuven, Leuven, Belgium; 3 Laboratory of Molecular Biology, Onze Lieve Vrouw Hospital, Aalst, Belgium; University of Hong Kong, China

## Abstract

Molecular surveillance of HRSV in Belgium for 15 consecutive seasons (1996–2011) revealed a shift from a regular 3-yearly cyclic pattern, into a yearly alternating periodicity where HRSV-B is replaced by HRSV-A. Phylogenetic analysis for HRSV-A demonstrated the stable circulation of GA2 and GA5, with GA2 being dominant over GA5 during 5 consecutive seasons (2006–2011). We also identified 2 new genotype specific amino acid mutations of the GA2 genotype (A122 and Q156) and 7 new GA5 genotype specific amino acid mutations (F102, I108, T111, I125, D161, S191 and L217). Several amino acid positions, all located in the second hypervariable region of HRSV-A were found to be under positive selection. Phylogenetic analysis of HRSV-B showed the circulation of GB12 and GB13, where GB13 represented 100% of the isolated strains in 4 out of 5 consecutive seasons (2007–2011). Amino acids under positive selection were all located in the aminoterminal hypervariable region of HRSV-B, except one amino acid located in the conserved region. The genotype distribution within the HRSV-B subgroup has evolved from a co-circulation of multiple genotypes to the circulation of a single predominant genotype. The Belgian GB13 strains circulating since 2006, all clustered under the BAIV branch and contained several branch specific amino acid substitutions. The demographic history of genotypes GA2, GA5 and GB13 demonstrated a decrease in the total GA2 and GA5 population size, coinciding with the global expansion of the GB13 population. The emergence of the GB13 genotype resulted in a newly established balance between the predominant genotypes.

## Introduction

Human respiratory syncytial virus (HRSV) is the most important viral agent causing serious lower airway infections in children less than 2 years old. HRSV can be divided into two subgroups, HRSV-A and HRSV-B and these subgroups harbour several genotypes, which represent clusters of co-circulating strains [1]–[7]. Viruses of the two antigenic groups commonly produce epidemics and annual epidemics that are characterized by the circulation of several genotypic strains. Genotyping of HRSV strains have historically been based on sequence data of the variable G glycoprotein [3]. The G protein has been shown to be the most divergent between HRSV-A and B subgroups with 67% identity on the nucleotide level and 53% similarity on the deduced amino acid level [8]. In addition, the G protein is one of the targets of neutralizing antibodies and continues to incorporate mutations due to existing immunological pressure [9]–[12]. For genotyping purposes, the hypervariable ectodomain of this protein has been selected as a reliable region for the entire G gene variability [3], [5]. This carboxyterminal domain encloses a first variable region starting at nucleotide position 284–459 for HRSV-A and 194–459 for HRSV-B. This domain is followed by a conserved cystein cluster and a second variable region located at nucleotide 649–918 for HRSV-A and nucleotide 652–921 for HRSV-B [8], [11], [13]. In 1998, Peret and coworkers defined HRSV genotypes based on the topology of phylogenetic trees of HRSV variants. For HRSV-A the genotypes GA1, GA2, GA3, GA4 and GA5 were identified with intergenotypic differences that ranged from 10–28% at the amino acid level. For HRSV-B, GB1 to 4 were distinguished and intergenotypic differences at the amino acid level ranged from 7–19% [3]. Previous genotyping efforts in Belgium introduced a genotype classification based on a gene segment of 629 bp for HRSV-A and 724–762 bp for HRSV-B comprising both hypervariable regions and the conserved region [6], [7]. Phylogenetic analyses have led to the distinction of 6 clusters that were assigned to genotypes BE/A1, GA1 to GA5, within the HRSV-A subgroup and GB1 to GB13 genotypes were identified within subgroup B. The genotype assignment of HRSV-A and –B strains was partially based on the genotype ascription of previous strains [3]–[5], [14] The GB13 genotype is characterised by a 60-nucleotide duplication and 6- nucleotide deletion and corresponds to the BA genotype described by Trento and co-workers [15].

Previous HRSV epidemics studied in Belgium showed that 2 subgroup A dominant seasons were followed by a subgroup B dominant season. Although it is difficult to explain the periodicity of the subgroup dominance, the dominant circulation of one subgroup over the other most likely results from the interplay of the pre-existing immunity in the community and the genetic and antigenic properties of the HRSV virus to evade the immune response. Over the past decades (1984–2006), the genotype circulation in Belgium was dynamic with circulation of BE/A1, GA1, GB1, GB2, GB3, GB4, GB5 and GB7 in the eighties and nineties [16]. These genotypes disappeared out of the population and were replaced by the predominant circulation of GA2, GA5, GB12 and GB13 genotypes.

In this study, HRSV seasonal epidemic dynamics in Belgium were monitored over a five-year period (2006–2011) investigating subgroup patterns and stability of genotypes previously circulating in Belgium. This epidemiological data (2006–2011) added to the previous epidemiological data obtained during 10 epidemic seasons (1996–2005), and allowed the interpretation of the HRSV dynamics over 15 consecutive years in Belgium [16]. Further, the genetic variability, phylogenetic relatedness and demographic history of HRSV-A and -B strains were investigated by mapping genetic diversity, performing selective pressure analysis and coalescent analysis that demonstrated the evolution of the virus population size over time.

## Materials and Methods

### Sample Collection

One-thousand-fifty-nine nasopharyngeal samples (NPS) from patients experiencing respiratory infections were collected during 5 HRSV epidemic outbreaks (2006/2007 till 2010/2011). Samples were acquired from patients invoking medical attention in the hospital for respiratory tract infection. Nine-hundred-ninety-three were obtained from the University Hospital of Leuven and 66 from the O.L.V. Hospital in Aalst. Both hospitals are located in Flanders and are situated 60 km from each other. All samples were HRSV positive because they had been previously tested positive by RSV antigen test (BinaxNOW RSV test, Medical Innovations Inc., Ireland), ‘in house’ PCR or qPCR before arriving at the laboratory. Nine-hundred-seventy-four samples were successfully subgrouped (Table 1) by means of qRT-PCR.

**Table 1:** Subgroup dominance per epidemic season.

Epidemic season	No. RSV positive samples	No. (%) of samplestyped*	No. (%) of HRSV infections*		
			HRSV-A	HRSV-B	HRSV-A & -B
2006/2007	128	127 (99.2)	9 (7.0)	111 (86.7)	7 (5.5)
2007/2008	140	126 (90.0)	101 (72.1)	17 (12.1)	8 (5.7)
2008/2009	252	231 (91.6)	50 (19.8)	149 (59.1)	32 (12.7)
2009/2010	402	389 (96.8)	271 (67.4)	114 (28.4)	4 (1.0)
2010/2011	137	101 (73.7)	84 (61.3)	17 (12.4)	0 (0.0)

* Percentages are calculated to the total number of RSV positive samples that were typed.

### RNA Extraction

Viral RNA was extracted from 140 µl of NPS by using the QIAmp viral RNA mini kit (Qiagen, Westburg, The Netherlands). RNA was extracted according to the manufacturers instructions and eluted in 60 µl elution buffer.

### Subgrouping

A multiplex qRT-PCR with a subgroup specific primer and probeset for HRSV-A and –B as described previously [16] was used to identify the subgroup of the virus strain.

### RT-PCR

The carboxyterminal region of the G protein was amplified by using the One-Step RT-PCR (Qiagen, Westburg, The Netherlands). The HRSV-A forward and reverse primer, G267FW and F164RV and the HRSV-B forward and reverse primer BGF and BGR separately to polyacrylamide gel electrophoresis and visualised under UV light by staining with ethidium bromide.

### Nucleotide Sequencing

The amplified PCR products were purified using the innuPREP PCRpure kit (Analytik Jena, Germany) and eluted in 20 µl elution buffer. Cycle sequencing was performed in forward and reverse direction using the ABI PRISM Big Dye Termination Cycle Sequencing Ready Kit (Applied Biosystems). In addition to the PCR amplification primers, the G516R (5′-GCTGCAGGGTACAAAGTTGAAC-3′) and G284F (5′-ACCTGACCCAGAATCCCCAG-3′) for HRSV-A and BGF3 (5′-AGAGACCCAAAAACACYAGCCAA-3′) and BGR3 (5′-ACAGGGAACGAAGTTGAACACTTCA-3′) primers for HRSV-B were used to assure complete consensus sequence of the amplicon. Sequence data was generated on the ABI3130xl Genetic Analyzer (Applied Biosystems). Sequences were manually edited and multiple sequence alignments were generated using the Clustal X 2.0.12 version [17]. Identical nucleotide sequences were identified by the DAMBE software version 4.2.13 [18]. Pairwise distances were calculated between unique sequences in MEGA5 [19]. Sequences were deposited in GenBank under the accesion numbers [JX645776-JX645982].

### Phylogenetic Analysis

We generated sequences of 629 bp and 724–762 bp for HRSV-A and HRSV-B respectively, to investigate the phylogenetic relationships between viral strains. Phylogenetic trees were constructed by the Neigbour Joining method using the MEGA5 software [19]. Bootstrap values were calculated based on 1000 replicates.

### Selective Pressure Analysis

We used a random effects likelihood (REL) approach based on a codon substitution model that allows for variation both in non-synonymous (*dN*) and synonymous rates (*dS*) to detect the selective pressure at an individual site in the G gene. This method fits a general bivariate distribution of *dN* and *dS* substitution rates across sites (each composed of three classes) and then infers the class to which each individual site belongs. The REL approach was applied using the HyPhy program and positive selected sites were detected by using empirical Bayes methods. Bayes factors >20 expressed as Log [BF{NS|S >1|S}] were used as a cut-off to identify positively selected sites.

### Bayesian Skyride Analysis

To estimate the effective population size dynamics through time we employed the Bayesian skyride approach as a flexible coalescent model [20]. The Bayesian skyride achieves temporal smoothing of the effective population size by exploiting Gaussian Markov random fields in a Bayesian framework implemented in the BEAST software version 1.7 [21]. We compiled a dataset for HRSV-A based on the GA2 and GA5 genotyped strains available in GenBank (Table S1) and the Belgian isolates sequenced in this study. The nucleotide sequence covered a 264 bp fragment of the second hypervariable region. For HRSV-B, sequences designated as GB13 genotype were used. Here, we used the same dataset as published by Trento and co-workers with in addition the Belgian sequence data from the epidemic seasons 2006–2011 (Table S2) [22]. For these analyses a nucleotide sequence alignment of 330 bp, comprising the second hypervariable region of the HRSV-B subgroup was employed. Sequences with a known year of isolation were included in the datasets and for the sequences with an unknown circulation date; the year of isolation was estimated [23].

## Results

### Age Distribution

**Figure 1:**
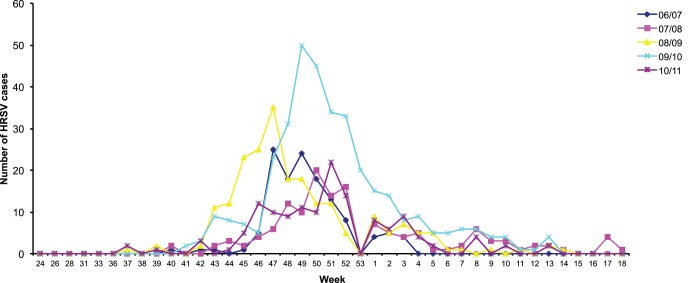
Seasonality of HRSV in Belgium (2006–2011).

The major population groups afflicted by HRSV infections are babies under the age of 12 months (81.5%), followed by patients older than 24 months and infants between 13 and 24 months. When looking at the age group older than 24 months in more detail, a diversification between several subpopulations can be made. This group consisted of 56.4% toddlers (<5 years), 23.1% children (5><13 years), 5.1% adolescents (13><26 years), 6.4% adults (28><60 years) and 6.4% elderly (65 years and older). In the group of adolescents, 4 patients were being followed at the paediatric oncology division and one patient had a history of paediatric immune deficiency. Regarding the general number of patients during 15 epidemic seasons in Belgium, the number of infected patients during the 2009/2010 season is remarkably high (Figure 1). During this season, the pandemic swine influenza virus (H1N1) was emerging worldwide, possibly alarming parents for the manifestation of respiratory infections in their children (Figure 1). This vigilance may have resulted in patients seeking medical attention much faster.

### Patients with Multiple and Prolonged HRSV Infections

Fourteen paediatric patients experienced two or three HRSV infections within the timeframe of 5 epidemic seasons (2006/2007–2010/2011) (Table 2). In 8 of the 14 cases the secondary infection was caused by a strain of the homologous subgroup: 4 A-A, 3 B-B and 1 A&B-A&B. Five patients were infected by a strain of the heterologous subgroup: 1 A-B, 3 B-A and 1 B- A&B co-infection. One patient encountered a HRSV infection during 3 consecutive epidemic seasons (07/08, 08/09 and 09/10) of which the second infection was caused by the heterologous subgroup. The third infection was induced by a strain of the homologous subgroup, compared to the primary infection. For 3 patients, the secondary infection was caused by a strain of the same genotype. The homologous A-A reinfection in table 2 was also a homologous GA2 genotype reinfection. Sequence comparison at the amino acid level revealed A113T, A130I, Q142L, P234L, E240K and N273Y alterations in the G protein when comparing the 2009 to the 2011 strain. Two patients were re-infected by the homologous GB13 genotype. Sequence comparison of the HRSV-B G protein revealed the incorporation of several amino acid substitutions. For the patient with a primary infection in 2006 and a secondary infection in 2008, the amino acid substitutions S138T, R153K, V171F, Q180R, N204R, K205E, A251V, T256I, S267L and L277S were observed. For the second patient, initially infected in 2008 and re-infected in 2010, the amino acid changes R98K, L124S, K136R, T138S, R153K, L219P, I256T, E261G, L267S and L277S were present.

**Table 2:** HRSV re-infections.

No. of HRSV re-infections						
A–A	A–B	B–B	B–A	AB–AB	B–A&B	B–A–A
4 (09/10–10/11)**	1 (07/08–08/09)	1 (06/07–08/09)*	1 (07/08–07/08)	1 (06/07–08/09)	1 (06/07–07/08)	1 (07/08–08/09–09/10)
		1 (08/09–08/09)	2 (08/09–09/10)			
		1 (08/09–10/11)*				

* Primary and secondary infections were caused by both strains of GB13 genotype **For 1 of the 4 re-infected patients, primary and secondary infections were caused by strains of GA2 genotype. For the other reinfections, genotypes of the primary and/or secondary strain are undetermined since G gene nucleotide sequence was not available. Epidemic season of primary, secondary or tertiary infection are indicated between brackets. All secondary infections occurred within 2 to 24 months. The tertiary infection occurred after 11 months (B–A–A).

In 2 patients, infected during the 2009/2010 season, a prolonged virus shedding was observed. A 2-month-old boy was infected with HRSV-A and nasopharyngeal samples remained positive for 2 months (December 2009 till February 2010). A 6-month-old girl was infected by HRSV-B and respiratory samples were positive from January 2010 till the end of March 2010. The medical history of these patients indicated that they were under attention of the oncology division of the hospital and most likely were suffering from immunological failure.

### Seasonality

HRSV seasons in Belgium traditionally set off in the fall (October) and can last till early spring (March) (Figure 1). Usually, an epidemiological peak is reached around week 50 in the winter. Remarkably, the epidemiological season of 2008/2009 was characterised by an early onset and an advanced epidemiological peak in week 47. Epidemic outbreaks have a mean duration of approximately 22 weeks.

### Subgroup Dominance

Subgrouping by means of qRT-PCR demonstrated a yearly alternating dominance between HRSV-B and HRSV-A during the epidemic season of 2006/2007 till 2009/2010 (Table 1). HRSV monitoring over 15 consecutive years has indicated a recurrent subgroup pattern of AAB during 9 out of 15 outbreaks (1996–2005) (Table 3). This temporal periodicity was replaced by an AB sequential repetition, in the four epidemics following (2006–2010). The latter season was followed by a second HRSV-A dominant season 2010/2011.

**Table 3:** Genotype distribution during 15 consecutive epidemic seasons.

Epidemicseason	No (%) of HRSV genotyped isolates																				
	GA2	%	GA5	%	GB2	%	GB3	%	GB6	%	GB8	%	GB10	%	GB11	%	GB12	%	GB13%	%	Subgroupdominance
1996/1997	1 (33)	8	**2 (67)**	**17**			1 (11)	8	**3 (33)**	**25**			**3 (33)**	25	1 (11)	8	1 (11)	8			A
1997/1998	**7 (70)**	**32**	3 (30)	14							3 (25)	14			1 (8)	4	**8 (67)**	**36**			A
1998/1999	**3 (60)**	**17**	2 (40)	12	2 (17)	12											**10 (83)**	**59**			B
1999/2000	7 (30)	15	**20 (70)**	**43**			1 (5)	2	3 (16)	7			1 (5)	2	5 (26)	11	**7 (37)**	15	2 (11)	4	A
2000/2001	9 (41)	28	**13 (59)**	**40**			4 (40)	13							2 (20)	6	**4 (40)**	**13**			A
2001/2002	**3 (75)**	7	1 (25)	2			1 (3)	2					1 (3)	2	9 (24)	22	1 (3)	2	**25 (68)**	**61**	B
2002/2003	2 (40)	5	**3 (60)**	**7**			1 (3)	3					3 (9)	8			**19 (54)**	**47**	12 (34)	30	A
2003/2004			**10 (100)**	**53**															**9 (100)**	**47**	A
2004/2005	**3 (100)**	**18**																	**14 (100)**	**82**	B
2005/2006	2 (29)	22	**5 (71)**	**56**															**2 (100)**	**22**	A
2006/2007	**11 (79)**	**11**	4 (29)	4													10 (12)	10	**76 (88)**	**75**	B
2007/2008	**60 (82)**	**66**	13 (18)	14															**17 (94)** *	**19**	A
2008/2009	**15 (75)**	**19**	5 (25)	6															**57 (100)**	**74**	B
2009/2010	**70 (100)**	**65**																	**38 (100)** *	**35**	A
2010/2011	**48 (98)**	**80**	1 (2)	2															**11 (100)**	**18**	A

* 1 strain unclassified.

Dominant genotypes are indicated in bold. #Numbers between brackets indicate the percentage of each genotype within the subgroup. % Column shows the relative proportion of each genotype to the total number of sequenced strains in that epidemic season.

### Phylogenetic Analysis HRSV-A

**Figure 2:**
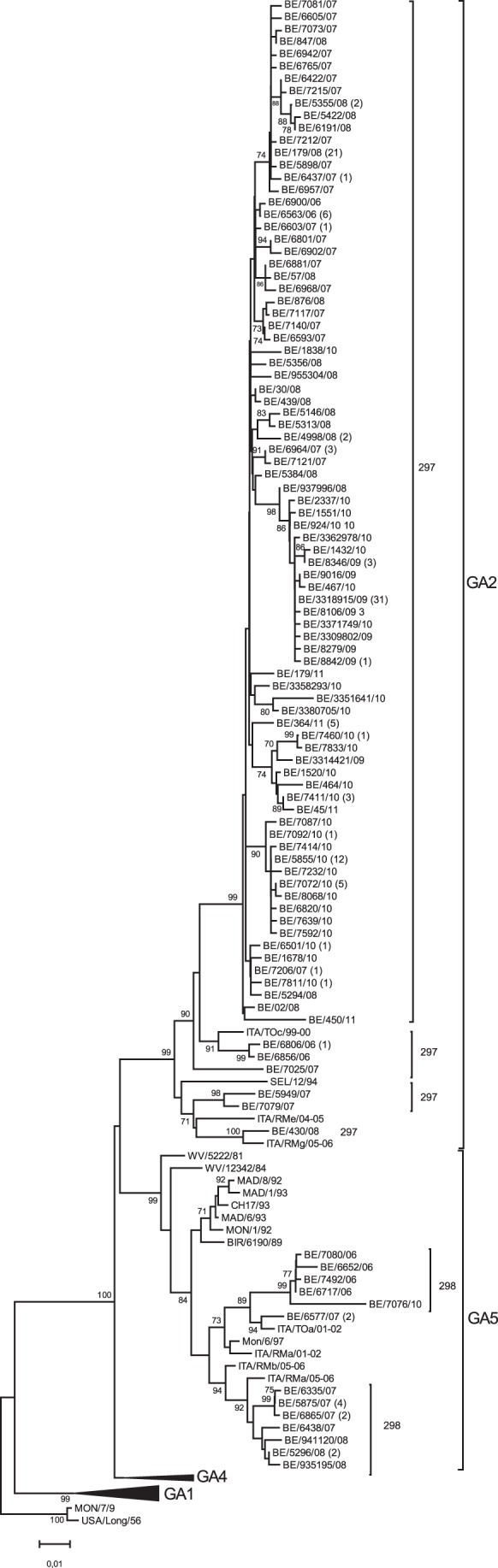
Phylogenetic tree of HRSV-A strains. The phylogenetic tree was constructed based on a 624-nucleotide fragment of the G protein, consisting of the two hypervariable regions. The nucleotide sequences of the Belgian strains isolated during 5 consecutive epidemic seasons (2006–2011) were compared with reference strains from Italy (ITA), Madrid (MAD), Montevideo (MON), West Virginia (WV) and Birmingham (BIR). The outgroup is represented by the Long strain. Bootstrap values are indicated at the internal nodes and are calculated for 1000 replicates by using the neighbour joining method. Only bootstrap values >70 are shown. The numbers between brackets are the number of strains identical to one shown in the tree. The amino acid lengths predicted for the G protein are indicated next to the Belgian isolates. For both the GA2 and GA5 genotypes, a strong temporal clustering of the strains per epidemic season is observed.

A total of 227 (44.2%) out of 515 HRSV-A strains were sequenced. One hundred and two sequences represented unique strains and the remaining 125 strains were found identical to other circulating strains. Clustering within the subgroup A with previous genotyped strains [3], [5] demonstrated the circulation of the GA2 and GA5 genotypes (Figure 2), with a clear dominance of GA2 over GA5 (Table 3). Belgian HRSV-A sequences isolated between 2006 and 2011 diverged in a range of 0.2–14.4% at the nucleotide level and in a range of 0.5–25.5% at the amino acid level. The deduced amino acid sequence of these strains showed a G protein of 297 amino acids within the GA2 genotype and a G protein of 298 residues within the GA5 genotype (Figure 2).

### Genotype Specific Amino Acid Mutations within Subgroup A

GA2 and GA5 are characterised by genotype specific amino acid mutations [24], [25]. All Belgian GA2 strains had the previous reported T269 and S289 amino acid substitution (Figure S1). The amino acid mutations A225, N250, S251, T274, I279, I295 and D297 were recognized in the second hypervariable region of the GA5 classified strains. Since we sequenced the nearly complete carboxyterminal ectodomain, including the first hypervariable region and the conserved region, we were able to recognize several other genotype specific amino acid mutations. GA2 strains were characterised by the A122 and Q156 amino acids. Botosso and colleagues report the L215P, R244Lis, H266Y, D297K and STOP298W mutation, fixed in the GA2 genotype. All Belgian GA2 strains isolated between 2006 and 2011 retained or mutated back to the original amino acid except for residue D297K [26]. We were able to differentiate GA5 from GA2 by the presence of F102, I108, T111, I125, D161, S191 and L217 at the amino acid level.

### Selective Pressure Analysis HRSV-A

Amino acids located at positions 237 (log BF = 4.4), 238 (log BF = 4.5), 244 (log BF = 5.06) and 262 (log BF = 5.03) were identified to be under diversifying selection. These positive selected sites are all located in the second hypervariable region of the G protein. None of the positive selected sites were serines or threonines, but several different amino acids were found at sites under positive selection: D/N/Y/H at position 237, T/P/F/I at position 238, R/G at position 244 and E or K at position 262.

### Phylogenetic Analysis HRSV-B

**Figure 3:**
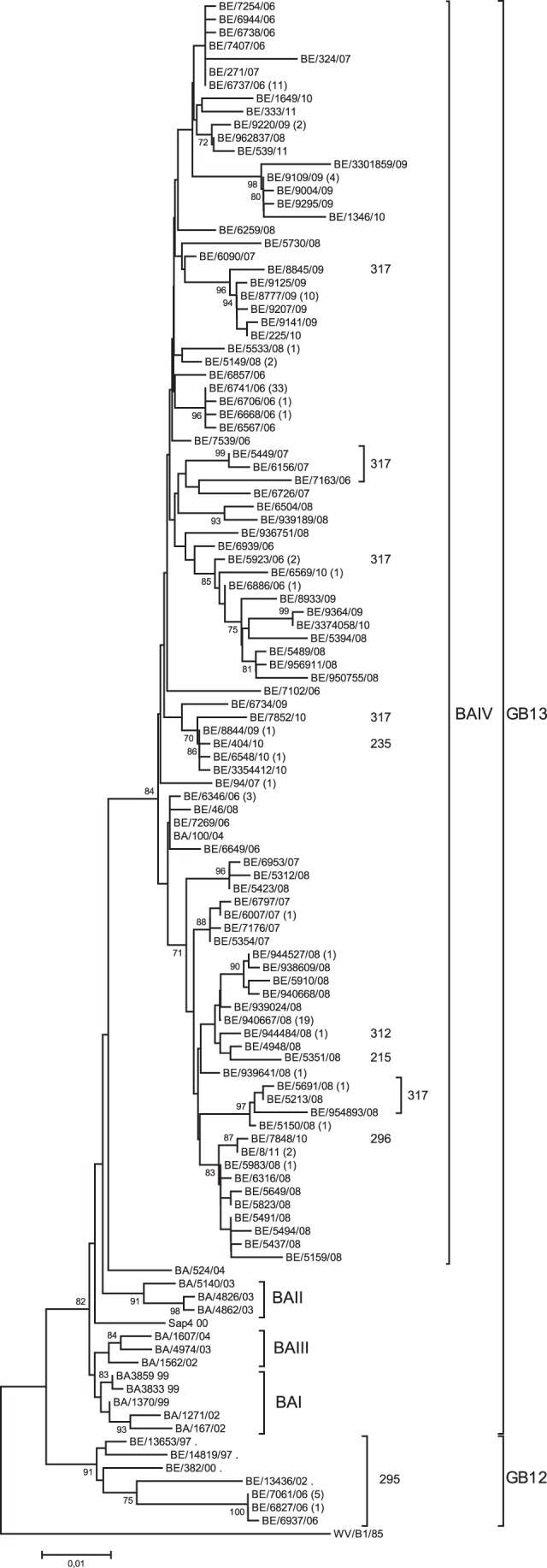
Phylogenetic tree of HRSV-B strains. The phylogenetic tree was constructed based on a 708–762-nucleotide fragment of the G protein, consisting of the two hypervariable regions. The nucleotide sequences of the Belgian strains isolated during 5 consecutive epidemic seasons (2006–2011) were compared with reference strains from Buenos Aires (BA), Belgium (BE) and South Africa (SA). The outgroup is represented by the WV/B1/85 strain. Bootstrap values are indicated at the internal nodes and are calculated for 1000 replicates by the neighbour joining method. Only bootstrap values >70 are shown. The numbers between brackets are the number of sequences identical to the sequence shown in the tree. The amino acid lengths predicted for the G protein are indicated next to the Belgian isolates. The isolates with no predicted G protein length indicated consisted of 310 residues.

**Figure 4:**
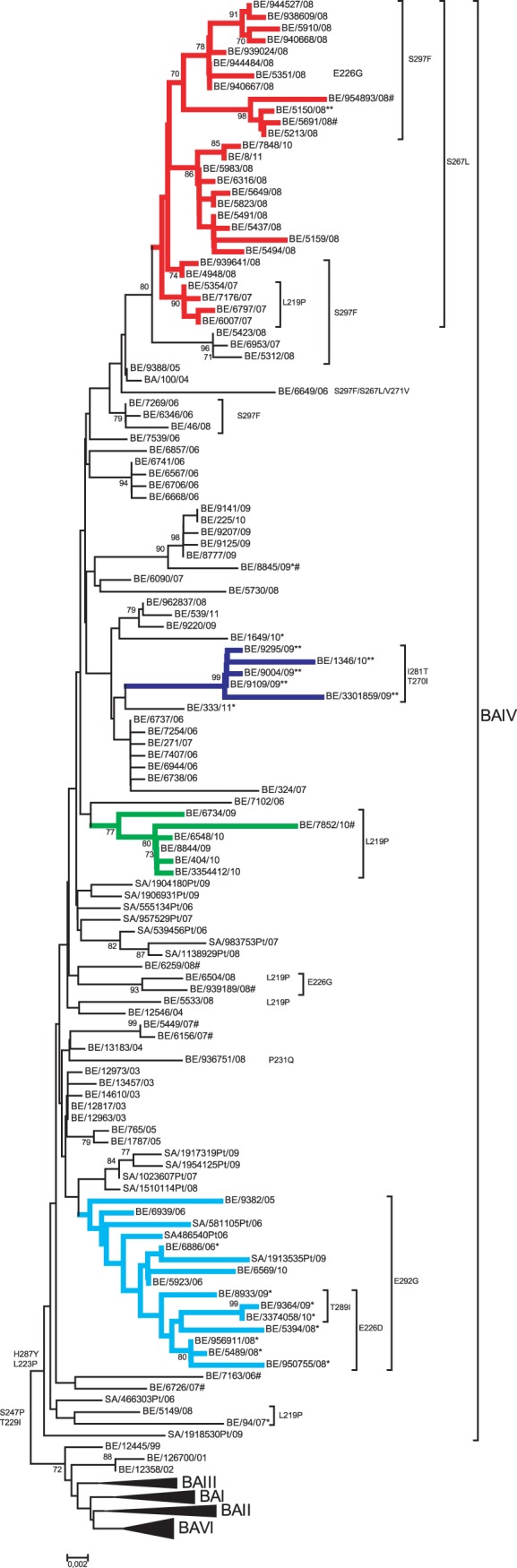
Phylogenetic analysis of HRSV-B GB13 strains designated to the BAIV lineage. The phylogenetic tree was constructed based on a 762-nucleotide fragment of the G protein, consisting of the two hypervariable regions. The nucleotide sequence of the GB13 designated strains were compared to strains from Buenos Aires (BA) and South Africa (SA) and assigned to branches of GB13. The numbers at the internal nodes represent bootstrap values, determined for 1000 iterations by the neighbour joining method. Only bootstrap values >70 are indicated. The amino acid substitutions that originate the main branches are indicated at the left of the nodes. Based on the presence of a certain amino acid position, several clusters were diversified. The red cluster groups the strains carrying the S267L substitution. The strains of the dark blue cluster share the I281T and T270I substitution. The cluster indicated in green groups the sequences with the L219P substitution. The turquois coloured branch indicates the sequences with the 2292G substitution. Within this cluster, sublineages characterised by the E226D mutation and T289I mutation can be distinguished. Brackets group strains with amino acid substitutions associated with the origin of new sublineages of the GB13 genotype. Strains with termination codon different from Q310STOP are indicated by #. Strains that do not have the H287Y substitution are indicated by single *. Strains indicated with **have the T270I substitution.

The partial G gene sequence (nt 177 to 900 according to reference strain WV/B1/85 accession number AF013254) of 213 (52.7%) out of 408 subgroup B isolates was determined and 105 sequenced strains were unique. One-hundred-and-eight sequences were identical to other circulating strains shown in Figure 3. Belgian isolates collected during the epidemic seasons of 2006 till 2011 had pairwise distances in the range of 0.1–7.3% at the nucleotide level and differed in a range of 0.4–10.8% at the amino acid level. Phylogenetic analysis demonstrated the circulation of two genotypes: GB12 and GB13. Two isolates remained unclassified (BE/6904/07 and BE/8773/09) (data not shown). The GB13 genotype is the predominating genotype of the circulating strains during the past 5 HRSV epidemic seasons (Figure 3). All GB13 strains circulating from 2006/2007 till 2010/2011 were circulating under the BAIV lineage (Figure 4).

### Amino Acid Analysis of HRSV-B

Protein lengths of 215, 235, 295, 296, 310, 312 and 317 residues were predicted based on the deduced amino acid sequence of the HRSV-B isolates (Figure S2). Remarkably, in all 5 seasons, the G protein length of 310 amino acids was dominant. The G protein polymorphisms result from genetic variation created by the presence of nucleotide mutations, a 6-nucleotide deletion, a 60-nucleotide duplication and the alternating use of three different stop codons (Figure S2). In addition to this genetic variety, we report a new 6-nucleotide insertion (GAAAAA) at nucleotide position 678 that was identified in two isolates (BE/950858/08 and BE/944484/08) clustering in the GB13 genotype, during the epidemic season of 2008/2009 (Figure S2). This in-frame-insertion is located in-between the 6-nucleotide deletion and the 60-nucleotide duplication and codes for the amino acids glutamine acid and lysine. In these strains the first stop codon was terminating and an amino acid length of 312 residues was predicted for the G glycoprotein. For 2 isolates, a nucleotide mutation led to the premature introduction of a stop codon resulting in a truncated G protein at the carboxy-terminal end. For isolate BE/404/10, the mutation AAG→TAG was responsible for a predicted protein length of 235 amino acids. The mutation AAA→TAA introduced a stop codon at amino acid position 216, resulting in a shortened G protein of BE/5351/08. For the BE/7848/10 strain, the presence of the 6-nucleotide-deletion, 60-nucleotide-duplication and CAA→TAA terminating mutation resulted in the amino acid length of 296 residues. Within the GB13 genotype, predicted protein lengths of 310 and 317 amino acids can be explained by the use of the first and third stop codon, respectively. Two strains, BE/6907/07 and BE/8773/09, had distinct predicted protein lengths of 295 and 299 residues resulting from second and third stop codon usage. The amino acid length of 295 amino acids was associated with the GB12 genotype.

### Genotype Specific Amino Acid Mutations within Subgroup B

All Belgian GB13 strains were characterised by the T229I and S247P substitution (Figure 4) and were classified as BA-IV based on clustering with isolates from Buenos Aires [22], [27] and South Africa [28]. All Belgian isolates contained the characteristic BA-IV H287Y substitution except for BE/6886/06, BE/8933/09 BE/9364/09 BE/3374058/10 BE/5394/08 BE/5489/08 BE/956911/08 BE/950755/08 and BE/94/07, BE/8845/09, BE/1649/10 and BE/333/11 (indicated in Figure 4 by *). However, several strains of the BA-IV branch (BE/6548/10, BE/3354412/10, BE/404/10, BE/884/09, BE/7852/10, BE/6734/09, BE/5533/08, BE/6504/08, BE/5149/08, BE/94/07, BE/6797/07, BE/6007/07, BE/7176/07 and BE/5354/07) also contained the BA-II associated L219P substitution. The BE/936751/08 strain contained the P231Q amino acid substitution associated with BA-V. The T270I substitution linked with branches BA-III to -V was also detected in several isolates (BE/5150/08, BE/9004/09, BE/1346/10, BE/9295/09, BE/9109/09 and BE/3301859/09).

Within the BA-IV branch, 6 additional substitutions (L223P, STOP316Q, Q268L/P, E292G, Q313STOP and V271A) were associated with the designation of subbranches: BAIVa, BAIVb, MAD-II, MAD-III, INDIA and BRAZIL [22]. The L223P substitution was found in all Belgian BAIV strains except for BE/333/11 and BE/5437/08. The Q268L/P substitution was not found in the Belgian GB13 isolates. The E292G substitution was found in a cluster of 12 strains. All Belgian strains had the Q313STOP mutation with the exception of BE/7852/10, BE/5691/08, BE/5213/08, BE/954893/10, BE/8845/09, BE/8923/06, BE/5449/07, BE/6156/07, BE/7163/06 and BE/6726/07. The V271A substitution, located in the duplicated region was also frequently detected, but not in the cluster of 12 strains that also had the E292G substitution and BE/1649/10, BE/6649/06 BE/6504/08, BE/939189/08, BE/6259/08, BE/7163/06 and BE/6726/07.

Recently, Dapat and colleagues suggested a reclassification of the BAIV branch, different from the subclassification proposed by Trento and co-workers, into BAIV, BA7, BA8 and BA9 [29]. They also reported the identification of a new BA10 branch. The BA7 branch was correlated to E226G and L223P substitution. Three isolates BE5351/08, BE/6504/08 and 939189/08 contained the E226G mutation.

The S267L substitution is shared by a cluster of 28 strains (indicated in red in Figure 4). Eighteen of these 28 strains also had the S297F substitution. In addition, 7 strains (BE/5423/08, BE/6953/07, BE/5312/08, BE/46/08, BE/7269/06, BE/6346/06 and BE/6649/06) not clustering in the S267L group, also contained the S297F substitution. The I281T is shared by a separate branch of 5 strains indicated in turquoise in Figure 4. These aforementioned substitutions (S267L, S297F and I281T) are characteristic for the BA9 branch. However, in our tree, the S267L substitution and the I281T substitution are shared by 2 separate clusters. BA10 is distinct by the substitutions E292G, E226D, T289I and S269P. Seven strains of the aforementioned E292G cluster also had the E226D amino acid substitution in common (indicated in blue Figure 4). Three strains of this cluster, also shared the T289I substitution. These 3 substitutions E292G, E226D and T289I have been linked to the branch BA10 [29].

### Selective Pressure Analysis HRSV-B

Positive selected sites were identified at amino acid positions T73, V76, T83, T92, T/I139, R/H151 and V171 (corresponding to strain WV/B1/85) with Log BF = 3.2. Remarkably, all these sites are located in the first hypervariable region of the G protein, with the exception of amino acid position 171, which is located in the conserved region. The threonines located at positions 76 and 171 were checked for O-glycosylation using the NetOglyc 3.1 program but the predicted potential of glycosylation was below the threshold, providing no evidence of O-linked sugar chains.

### Demographic History of GA2, GA5 and GB13 Genotypes Worldwide

**Figure 5:**
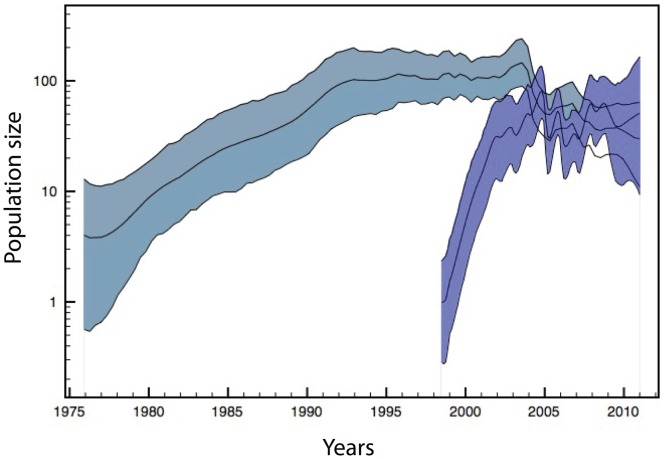
Bayesian skyride plot. Demographic history of HRSV-A genotypes GA2 and GA5 is represented in blue and the HRSV-B GB13 genotype is shown in purple. The population size (Neτ) is indicated on the y-axis and the x-axis demonstrates the time period in years. The inner line represents the median estimate and the coloured areas in blue or purple indicate the 95% highest posterior density.

The population size dynamics of GA2, GA5 and GB13 were estimated using Bayesian skyride analysis including strains isolated in different parts of the world. The combination of the GA2 and GA5 nucleotide dataset, encompassing the second hypervariable region of the G protein, resulted in the skyride plot for HRSV-A (Figure 5). This estimate of population size through time for HRSV-A was compared to the demographic history of GB13. The Bayesian skyline plots clearly demonstrated an expansion for the GB13 genotype after 1998. This expansion levelled of and was followed by a period of roughly constant population size. Interestingly, at the time point that the GB13 population size attained a relatively high level, the population size of the HRSV-A genotypes underwent a decrease. After this decrease, the total population size of GA2 and GA5 genotypes approximated the total population size of GB13 strains, balancing the population sizes of HRSV-A and –B stable genotypes.

## Discussion

Seasonal outbreaks of HRSV in Belgium have been documented since the epidemic season of 1982–1983 [6], [7], [16]. Annually, HRSV infections appear in October and reach a peak in December. Remarkably, during the season of 2008–2009, the peak incidence was observed in November. This earlier peak could not be explained by meteorological parameters such as temperature, humidity and the particulate matter concentrations in ambient air [30]. Next to these factors, human behavior e.g. indoor crowding and immunologic susceptibility e.g. level of maternal antibodies, contribute to the seasonal character of these outbreaks [31], [32]. Children below the age of 12 months represented 81.5% of the infected patients during the study. Here, we also observed that HRSV is an important pathogen in immunocompromised patients, resulting in prolonged virus shedding. A total of 14 re-infections were observed during the study period, where homologous subgroup (8/14) and heterologous subgroup (6/14) infections were detected (Table 2). For 3 homologous infections, secondary infection was caused by a strain of the homologous genotype. Several amino acid substitutions were detected between primary and secondary strains of the same genotype suggesting that genetic variability may have resulted in the escape to the immune response. Previous studies have reported that strain-specific and group-specific immunity waned after 7 to 9 and 2 to 4 months respectively, allowing repeated infections to be encountered [33], [34].

During 10 consecutive seasons (1996–2006), a regular 3-yearly cyclic pattern has been observed, where two HRSV-A dominant seasons alternated with one HRSV-B season. Since the HRSV season of 2005/2006, this has changed into a yearly alternating model where HRSV-A was replaced by HRSV-B in the subsequent season. However, the HRSV-A dominance during the 2010/2011 season introduced a second break of the subgroup pattern possibly initiating a new alteration in the subgroup periodicity. Overall, subgroup A strains (10/15) were dominating more seasons than subgroup B strains (5/15).

For HRSV-A, GA2 and GA5 are genotypes that have been circulating in the Belgian population since 1983–1984 [6]. For HRSV-B, a replacement of a circulation pattern of multiple genotypes (GB1-GB12) by the circulation of a single genotype GB13 has occurred in 2003 [7], [16]. For the GB13 gentoype, a strong temporal clustering of the strains per epidemic season is observed. The GB12 genotype was only circulating in the Belgian population during the epidemic season of 2006/2007. All the isolated GB13 strains belonged to the BAIV lineage. Other genotypes such as the GA1, GA4 and BE/A1 genotype circulated in the epidemic seasons of 1989/1990, 1992/1993 and 1986/1987, respectively but have disappeared out of the Belgian population. Also GB2, GB3, GB6, GB8, GB10 and GB11 have not been isolated after the epidemic season of 2002/2003. The GB13 genotype, first detected in Belgium in 2001/2002 has replaced all other genotypes and co-circulated with GB12 in the season 2002/2003 and 2006/2007. This gradual replacement of circulating genotypes by GB13 has been observed in different countries and GB13 represents 100% of the isolated HRSV-B strains after 2006 [25], [28]. Since 2006/2007, GA2 has been predominating over GA5, whereas the genotype distribution pattern of the previous seasons (1996/1997–2005/2006) demonstrated annual or biennial replacement of GA5 by GA2 or vice versa. GA2 and GA5 circulate worldwide and are recognized as stable genotypes [24], [25], [28], [35]–[40]. The select dominance of GA2 has also been observed in South Western China during epidemic seasons 2006–2009 [40] and Central and South America [36], Ireland in 2008/2009 [25] and South Africa in 2007–2008 [28]. The efficient circulation of these genotypes could not be explained by high genetic variability of the carboxy-terminal hypervariable region, since this region was remarkably conserved comparing strains isolated over several years [41]. Amino acid analysis of subgroup A strains suggested a predicted protein length of 297 residues for GA2 strains and a protein length of 298 amino acids for GA5 strains. In addition, we identified 2 new amino acid specific mutation of the GA2 genotype (A122 and Q156) and 7 new amino acid mutations (F102, I108, T111, I125, D161, S191 and L217) that differentiated the GA5 genotype from the GA2. Recently, it has been speculated that the variation in the amino-terminal variable region may be responsible for sustained virulence and may have allowed its prolonged circulation. Until now, the immunological importance of the amino-terminal variable region remains to be elucidated [41]. However, the identification of 4 amino acid positions (237, 238, 244 and 262) under positive selection, located in the second hypervariable region, is indicative of diversifying selective pressure acting in this gene region. Selective pressure by the immunological response has been described as one of the mechanisms that drive genetic variability of HRSV [42]. Strains that possess an asparagine at site 237 are potentially N-glycosylated. Glycosylation is an important hallmark of antigenicity of the virus, since it can mask or facilitate recognition by antibodies of the immune response [26]. The presence of an arginine at amino acid position 244 in isolates is associated with the loss of reaction with monoclonal antibodies [43]. For HRSV-B, 6 amino sites (73, 76, 83, 92, 139 and 151) were under positive selection and these amino acids were all located in the first hypervariable region of the G protein, except for amino acid 171, located in the conserved region. Immunologic pressure seems to act mainly in the first hypervariable region and the conserved region, which is remarkable. Not much is known about the location of strain specific epitopes, however, immunological pressure acting in the conserved region is notable. The amino acid found at a certain positive selected site varied between strains, indicating the occurrence of “flip-flop” reversions over time [26]. Further, the amino acid analysis of the HRSV-A G protein supports this because almost all the GA2 specific mutations reported by Botosso and co-workers reverted to their original amino acid in the Belgian strains. Also amino acid position 237 that is under positive selection was described to be subject of several flip-flop reversions. These flip-flop reversions are likely responsible for the loss of protective immunity that may have been evoked against key epitopes [26]. For HRSV-B, the reversion of amino acid at position 171 in the HRSV-B GB13 strain contributed to the re-infection with homologous genotype (Table 2).

The G protein of HRSV-B isolates is polymorphic which can be explained by the introduction of early stop codons, the alternating use of 3 stop codons, the presence of a 60-nucleotide duplication, 6-nucleotide deletion and a novel 6-nucleotide insertion. These 6 nucleotides are probably inserted due to polymerase stuttering during transcription or replication since these bases are flanked by a guanosine combined with an adenosine cluster (Figure S2). Remarkably, the first triplet of this insertion corresponds to the position of the 3-nucleotide-insertion that has been reported for 5 Belgian isolates during the epidemic season of 1995/1996 that clustered in the GB9 genotype. After this epidemic season, the 3-nucleotide-insertion has disappeared out of the Belgian population and now reappears, 14 epidemic years later, combined with a second triplet, in GB13 strains. Most likely, these insertions resulted from 2 separate mutational events. The polymorphism of HRSV-B isolates has led to the prediction of protein lengths of 215, 235, 295, 296, 310, 312 and 317 residues, with G proteins of 310 amino acids being predominant.

The GB13 genotype can be subdivided in several branches BAI to BAVI based on clustering with previous strains assigned to these branches [27], [28]. Belgian GB13 isolates circulating from 1999–2005 were classified under 5 branches BAI-IV and BAVI [22]. All the GB13 strains detected since 2006 belonged to the BAIV branch. The spread of the BAIV lineage is observed worldwide and suggests the successful transmission of the GB13 genotype.

Several amino acid substitutions located in the second hypervariable region of GB13 strains have been correlated with clusters within the GB13 genotype. However, some BAII, -III and –V specific amino acid substitutions were also detected in the Belgian BAIV strains. A subdivision of the BAIV branch into 6 categories, BAIVa, BAIVb, MAD-II, MAD-III, INDIA and BRAZIL, was proposed based on detection of additional substitutions and phylogenetic clustering [22]. In parallel, a different diversification of the BAIV branch was reported, where BAIV was re-classified as BAIV, BA7 to BA9 and a new genotype BA10 [29]. Although many amino acid substitutions were detected among the Belgian strains, it was not possible to correlate the amino acid substitutions to the (sub-) branches. Because no sequence data is available that covers the complete ectodomain of the G protein for strains of these subclassifications, their implementation by means of phylogenetic analysis was not possible.

GA2 and GA5 are genotypes that co-circulate worldwide, and the predominance of the GB13 genotype has been reported for several countries [28], [40]. The demographic history of the dominant HRSV-A genotypes and the predominant HRSV-B genotype, reconstructed through a Bayesian skyride analysis demonstrated a decrease in HRSV-A population size at the time the GB13 expansion was levelled off (Figure 5). As a consequence the HRSV-A population size was in balance with the HRSV-B population around the epidemic season of 2004–2005. The population size of GB13 is subsequently characterized by a roughly constant population size that overlaps with the epidemiological period where a yearly alternating subgroup dominance was observed in Belgium. This study is the first to demonstrate that the dissemination of the GB13 genotype may have led to a decreased total population size of the GA2 and GA5 genotypes worldwide resulting in a new equilibrium between of the total HRSV-A and –B virus population. The proportional circulation of these stable genotypes may be at the origin of the altered subgroup pattern. However, more studies need to be conducted to support this hypothesis.

Genetic mapping of the G protein gene remains important to identify new genetically diverse HRSV variants that may spread globally. This is illustrated by the identification of HRSV-B GB13 and the recently described HRSV-A ON1, two novel genotypes containing a 60-nucleotide and 72-nucleotide duplication in the second hypervariable region of the ectodomain [44]. The global spread of GB13 is documented while for HRSV-A, it needs to be awaited if the 72-nucleotide duplication will provide similar transmission success. In addition, investigation of the genetic diversity ensures the efficacy of the current vaccines and antiviral compounds in development. The global spread of novel genotypes may imply that the vaccines currently in development need to be adjusted [28].

## Supporting Information

Figure S1 The amino acid substitutions in the hypervariable regions of HRSV-A GA2 and GA5 genotypes. The amino acid substitutions in the hypervariable regions of HRSV-A GA2 and GA5 genotypes. Deduced amino acid alignment of the G protein gene from a selection of HRSV-A sequences isolated during 5 consecutive epidemic seasons (2006/2007–2010/2011). The alignment is shown relative to the reference strain AUS/A2/61 (M11486). The amino acid residues depicted start at position 85 to 299. The conserved region is located at amino acid position 164–176 with four cysteines at positions 173, 176, 182 and 186. Identical residues or identical stopcodon positions to the reference sequences are depicted as dots, and alternative used stop codons are indicated by asterisk. GA2 genotype specific amino acid mutations are indicated in yellow. GA5 specific amino acid mutations are indicated in grey.
(EPS)

Figure S2 G protein polymorphisms of HRSV-B strains. Schematic representation of the predicted G protein lengths for the Belgian HRSV-B isolates in accordance with the WV/B1/85 reference strain. The G protein ectodomain consists of two hypervariable regions separated by a central conserved region (amino acid positions 153 to 221). The conserved region consists of 4 cysteine residues located between amino acids 164 and 187 and are indicated by a dashed line. The underlined sequence represents the used termination codon and the total protein length is indicated at the end of each G protein. The 6-nucleotide deletion is shown as a V, where the deleted amino acids are indicated in top. The 60-nucleotide duplication region is visualised as a paired black box, with the duplicated sequence indicated above the box. The 6-nucleotide insertion is indicated as a little black box. The insertion of premature stopcodons are visualised by a dot (•) resulting in truncated G proteins.
(EPS)

Table S1 HRSV-A sequence data used in the coalescent analysis.
(DOCX)

Table S2 HRSV-B sequence data used in the coalescent analysis.
(DOCX)
