# Supplementary material for: Circulation of HRSV in Belgium: From Multiple Genotype Circulation to Prolonged Circulation of Predominant Genotypes
Source: PLoS One. 2013 Apr 5;8(4):e60416. doi: 10.1371/journal.pone.0060416 (PMC3618235; doi:10.1371/journal.pone.0060416)
Supplement: Table S1 — HRSV-A sequence data used in the coalescent analysis. (DOCX) [file pone.0060416.s003.docx]

**Table S1. HRSV-A sequence data used in the coalescent analysis**

| **RSV-A strain** | **Country** | **Isolation year** | **Accession number** | **Reference** |
| --- | --- | --- | --- | --- |
| 91087 | South Korea | 1991 | AF193304 | [1] |
| 91142 | South Korea | 1991 | AF193305 |  |
| 91399 | South Korea | 1991 | AF193307 |  |
| 92011 | South Korea | 1992 | AF193308 |  |
| 92308 | South Korea | 1992 | AF193309 |  |
| 92359 | South Korea | 1992 | AF193310 |  |
| 92415 | South Korea | 1992 | AF193311 |  |
| 93006 | South Korea | 1993 | AF193313 |  |
| 93057 | South Korea | 1993 | AF193314 |  |
| 94118 | South Korea | 1994 | AF193315 |  |
| 94191 | South Korea | 1994 | AF193316 |  |
| 95026 | South Korea | 1995 | AAF23740 |  |
| 95103 | South Korea | 1995 | AF193318 |  |
| 95118 | South Korea | 1995 | AAF23742 |  |
| 96308 | South Korea | 1996 | AF193322 |  |
| A/WI/629-9-2/07 | USA | 2007 | JF920048 | [2] |
| A/WI/629-DC9/08-09 | USA | 2008-2009 | JF920050 |  |
| A/WI/629-Q0198/10 | USA | 2010 | JF920055 |  |
| AL19376-1 | USA | 1994-1995 | AF233900 | [3] |
| AL19452-2 | USA | 1994-1995 | AF233901 |  |
| AL19556-3 | USA | 1994-1995 | AF348810 |  |
| CN1973 | Canada | 1994-1995 | AF233904 |  |
| CN2708 | Canada | 1994-1995 | AF233906 |  |
| MO55 | USA | 1994-1995 | AF233915 |  |
| Ar/1/97 | Argentina | 1997 | AF516121 | [4] |
| Ar/1/98 | Argentina | 1998 | AF516126 |  |
| Ar/2/97 | Argentina | 1997 | AF516122 |  |
| Ar/2/98 | Argentina | 1997 | AF516127 |  |
| Ar/3/97 | Argentina | 1997 | AF516123 |  |
| Mon/1/00 | Uruguay | 2000 | AF516128 |  |
| Mon/1/93 | Uruguay | 1993 | AF516107 |  |
| Mon/1/94 | Uruguay | 1994 | AF448498 |  |
| Mon/1/95 | Uruguay | 1995 | AF516108 |  |
| Mon/1/97 | Uruguay | 1997 | AF516117 |  |
| Mon/2/01 | Uruguay | 2001 | AF516136 |  |
| Mon/2/96 | Uruguay | 1996 | AF516111 |  |
| Mon/2/98 | Uruguay | 1998 | AF516125 |  |
| Mon/3/00 | Uruguay | 2000 | AF516130 |  |
| Mon/3/01 | Uruguay | 2001 | AF516137 |  |
| Mon/3/96 | Uruguay | 1996 | AF516112 |  |
| Mon/3/97 | Uruguay | 1997 | AF516119 |  |
| Mon/4/96 | Uruguay | 1996 | AF516113 |  |
| Mon/4/97 | Uruguay | 1997 | AF516120 |  |
| Mon/6/00 | Uruguay | 2000 | AF516133 |  |
| BA/2961/98 | Argentina | 1998 | AY667077 | [5] |
| BA/2964/98 | Argentina | 1998 | AY667078 |  |
| BA/3144/98 | Argentina | 1998 | AY667079 |  |
| BA/3771/99 | Argentina | 1999 | AY667080 |  |
| BA/3793/99 | Argentina | 1999 | AY667081 |  |
| BA/3892/99 | Argentina | 1999 | AY667083 |  |
| BA/3961/99 | Argentina | 1999 | AY667084 |  |
| BA/4940/00 | Argentina | 2000 | AY667086 |  |
| BA/4975/00 | Argentina | 2000 | AY667087 |  |
| BA/5046/00 | Argentina | 2000 | AY667089 |  |
| BA/5049/00 | Argentina | 2000 | AY667090 |  |
| BA/5066/00 | Argentina | 2000 | AY667091 |  |
| BA/5081/00 | Argentina | 2000 | AY667092 |  |
| BA/5948/01 | Argentina | 2001 | AY667093 |  |
| BA/5952/01 | Argentina | 2001 | AY667094 |  |
| BA/6091/01 | Argentina | 2001 | AY667095 |  |
| BA/6122/01 | Argentina | 2001 | AY667096 |  |
| BE/004/02 | Belgium | 2002 |  | [6] |
| BE/01/02 | Belgium | 2002 | DQ985124 |  |
| BE/617/98 | Belgium | 1998 | DQ985131 |  |
| BE/64/01 | Belgium | 2001 | AY343566 |  |
| BE/03/03 | Belgium | 2003 | DQ985096 |  |
| BE/10365/05 | Belgium | 2005 | DQ985103 |  |
| BE/11/01 | Belgium | 2001 | AY343611 |  |
| BE/11030/00 | Belgium | 2000 | AY343599 |  |
| BE/11091/00 | Belgium | 2000 | AY343601 |  |
| BE/11129/00 | Belgium | 2000 | AY343600 |  |
| BE/112/01 | Belgium | 2001 | AY343578 |  |
| BE/11584/01 | Belgium | 2001 | AY343645 |  |
| BE/11772/96 | Belgium | 1996 | DQ985117 |  |
| BE/11886/02 | Belgium | 2002 | DQ985105 |  |
| BE/11976/00 | Belgium | 2000 | AY343565 |  |
| BE/11996/00 | Belgium | 2000 | AY343607 |  |
| BE/1224/01 | Belgium | 2001 | AY343571 |  |
| BE/12243/96 | Belgium | 1996 | AY343647 |  |
| BE/12303/98 | Belgium | 1998 | DQ985127 |  |
| BE/12350/96 | Belgium | 1996 | AY343593 |  |
| BE/12511/96 | Belgium | 1996 | DQ985118 |  |
| BE/12752/02 | Belgium | 2002 | DQ985114 |  |
| BE/12808/97 | Belgium | 1997 | DQ985132 |  |
| BE/12971/04 | Belgium | 2004 | DQ985126 |  |
| BE/13024/02 | Belgium | 2002 | DQ985095 |  |
| BE/13083/99 | Belgium | 1999 | AY343551 |  |
| BE/13393/99 | Belgium | 1999 | AY343612 |  |
| BE/13412/99 | Belgium | 1999 | AY343646 |  |
| BE/13425/99 | Belgium | 1999 | AY343603 |  |
| BE/1343/01 | Belgium | 2001 | AY343574 |  |
| BE/1345/00 | Belgium | 2000 | AY343580 |  |
| BE/13462/99 | Belgium | 1999 | AY343643 |  |
| BE/13949/98 | Belgium | 1998 | DQ985110 |  |
| BE/14140/97 | Belgium | 1997 | DQ985123 |  |
| BE/14162/98 | Belgium | 1998 | DQ985106 |  |
| BE/14246/03 | Belgium | 2003 | DQ985088 |  |
| BE/14461/98 | Belgium | 1998 | DQ985088 |  |
| BE/14536/98 | Belgium | 1998 | AY343583 |  |
| BE/14808/98 | Belgium | 1998 | AY343609 |  |
| BE/14898/98 | Belgium | 1998 | AY343610 |  |
| BE/15471/97 | Belgium | 1997 | AY343594 |  |
| BE/15473/97 | Belgium | 1997 |  |  |
| BE/15739/97 | Belgium | 1997 | AY343616 |  |
| BE/16/00 | Belgium | 2000 | AY343558 |  |
| BE/1836/01 | Belgium | 2001 | AY343576 |  |
| BE/1936/00 | Belgium | 2000 | AY343569 |  |
| BE/1937/04 | Belgium | 2004 | DQ985094 |  |
| BE/2057/03 | Belgium | 2003 | DQ985129 |  |
| BE/2122/00 | Belgium | 2000 | AY343559 |  |
| BE/2355/97 | Belgium | 1997 | DQ985116 |  |
| BE/2363/98 | Belgium | 1998 | DQ985112 |  |
| BE/519/01 | Belgium | 2001 | AY343605 |  |
| BE/332/02 | Belgium | 2002 | AY343613 |  |
| BE/5445/02 | Belgium | 2002 | DQ985121 |  |
| BE/298/02 | Belgium | 2002 | DQ985102 |  |
| BE/8885/05 | Belgium | 2005 | DQ985130 |  |
| BE/901/01 | Belgium | 2001 | AY343563 |  |
| BE/9222/05 | Belgium | 2005 | DQ985089 |  |
| BE/9522/05 | Belgium | 2005 | DQ985099 |  |
| BE/9567/05 | Belgium | 2005 | DQ985100 |  |
| BE/9635/05 | Belgium | 2005 | DQ985098 |  |
| BE/9728/05 | Belgium | 2005 | DQ985101 |  |
| BE/9335/05 | Belgium | 2005 | DQ985097 |  |
| BE/30/08 | Belgium | 2008 |  | This study |
| BE/109/08 | Belgium | 2008 |  |  |
| BE/313/08 | Belgium | 2008 |  |  |
| BE/3314421/09 | Belgium | 2009 |  |  |
| BE/02/08 | Belgium | 2008 |  |  |
| BE/3351641/10 | Belgium | 2010 |  |  |
| BE/3380705/10 | Belgium | 2010 |  |  |
| BE/35/08 | Belgium | 2008 |  |  |
| BE/38/97 | Belgium | 1997 |  |  |
| BE/430/08 | Belgium | 2008 |  |  |
| BE/439/08 | Belgium | 2008 |  |  |
| BE/45/11 | Belgium | 2011 |  |  |
| BE/450/11 | Belgium | 2010 |  |  |
| BE/464/10 | Belgium | 2010 |  |  |
| BE/4998/08 | Belgium | 2008 |  |  |
| BE/5146/08 | Belgium | 2008 |  |  |
| BE/5296/08 | Belgium | 2008 |  |  |
| BE/5313/08 | Belgium | 2008 |  |  |
| BE/5356/08 | Belgium | 2008 |  |  |
| BE/5384/08 | Belgium | 2008 |  |  |
| BE/5422/08 | Belgium | 2008 |  |  |
| BE/57/08 | Belgium | 2008 |  |  |
| BE/5875/07 | Belgium | 2007 |  |  |
| BE/5898/07 | Belgium | 2007 |  |  |
| BE/5949/07 | Belgium | 2007 |  |  |
| BE/6422/07 | Belgium | 2007 |  |  |
| BE/6437/07 | Belgium | 2007 |  |  |
| BE/6438/07 | Belgium | 2007 |  |  |
| BE/6501/10 | Belgium | 2010 |  |  |
| BE/6563/06 | Belgium | 2006 |  |  |
| BE/6577/07 | Belgium | 2007 |  |  |
| BE/6603/07 | Belgium | 2007 |  |  |
| BE/6605/07 | Belgium | 2007 |  |  |
| BE/6652/06 | Belgium | 2006 |  |  |
| BE/6717/06 | Belgium | 2006 |  |  |
| BE/6765/07 | Belgium | 2007 |  |  |
| BE/6801/07 | Belgium | 2007 |  |  |
| BE/6806/06 | Belgium | 2006 |  |  |
| BE/6824/07 | Belgium | 2007 |  |  |
| BE/6856/06 | Belgium | 2006 |  |  |
| BE/6881/07 | Belgium | 2007 |  |  |
| BE/6883/07 | Belgium | 2007 |  |  |
| BE/6900/06 | Belgium | 2006 |  |  |
| BE/6902/07 | Belgium | 2007 |  |  |
| BE/6942/07 | Belgium | 2007 |  |  |
| BE/6957/07 | Belgium | 2007 |  |  |
| BE/696/08 | Belgium | 2008 |  |  |
| BE/6965/07 | Belgium | 2007 |  |  |
| BE/6968/07 | Belgium | 2007 |  |  |
| BE/6998/07 | Belgium | 2007 |  |  |
| BE/7073/07 | Belgium | 2007 |  |  |
| BE/7076/10 | Belgium | 2010 |  |  |
| BE/7079/07 | Belgium | 2007 |  |  |
| BE/7080/06 | Belgium | 2006 |  |  |
| BE/7081/07 | Belgium | 2007 |  |  |
| BE/7083/07 | Belgium | 2007 |  |  |
| BE/7087/10 | Belgium | 2010 |  |  |
| BE/7092/10 | Belgium | 2010 |  |  |
| BE/7121/07 | Belgium | 2007 |  |  |
| BE/7124/06 | Belgium | 2006 |  |  |
| BE/7206/07 | Belgium | 2007 |  |  |
| BE/7215/07 | Belgium | 2007 |  |  |
| BE/7232/10 | Belgium | 2010 |  |  |
| BE/7407/06 | Belgium | 2006 |  |  |
| BE/7411/10 | Belgium | 2010 |  |  |
| BE/7492/06 | Belgium | 2006 |  |  |
| BE/7557/10 | Belgium | 2010 |  |  |
| BE/758/03 | Belgium | 2003 |  |  |
| BE/7598/10 | Belgium | 2010 |  |  |
| BE/7639/10 | Belgium | 2010 |  |  |
| BE/7811/10 | Belgium | 2010 |  |  |
| BE/7833/10 | Belgium | 2010 |  |  |
| BE/7849/10 | Belgium | 2010 |  |  |
| BE/797/00 | Belgium | 2000 |  |  |
| BE/800/00 | Belgium | 2000 |  |  |
| BE/8034/10 | Belgium | 2010 |  |  |
| BE/8081/09 | Belgium | 2009 |  |  |
| BE/8107/09 | Belgium | 2009 |  |  |
| BE/822/00 | Belgium | 2000 |  |  |
| BE/8327/09 | Belgium | 2009 |  |  |
| BE/847/08 | Belgium | 2008 |  |  |
| BE/876/08 | Belgium | 2008 |  |  |
| BE/8860/09 | Belgium | 2009 |  |  |
| BE/935195/08 | Belgium | 2008 |  |  |
| BE/937996/08 | Belgium | 2008 |  |  |
| BE/938865/08 | Belgium | 2008 |  |  |
| BE/941120/08 | Belgium | 2008 |  |  |
| BE/944/00 | Belgium | 2000 |  |  |
| BE/944262/08 | Belgium | 2008 |  |  |
| BE/955304/08 | Belgium | 2008 |  |  |
| BE/1432/10 | Belgium | 2010 |  |  |
| BE/1520/10 | Belgium | 2010 |  |  |
| BE/1838/10 | Belgium | 2010 |  |  |
| BE/2337/10 | Belgium | 2010 |  |  |
| BE/1678/10 | Belgium | 2010 |  |  |
| BE/179/11 | Belgium | 2011 |  |  |
| BE/1/11 | Belgium | 2011 |  |  |
| Beijing/01/01 | China | 2001 | DQ289597 | [7] |
| Beijing/01/05 | China | 2001 | DQ289598 |  |
| Beijing/01/10 | China | 2001 | DQ289599 |  |
| Beijing/04/08 | China | 2004 | DQ289606 |  |
| Beijing/04/23 | China | 2004 | DQ289616 |  |
| Beijing/04/32 | China | 2004 | DQ289621 |  |
| Beijing/04/33 | South Korea | 2004 | DQ289622 |  |
| Beijing/04/34 | China | 2004 | DQ289623 |  |
| Beijing/04/42 | China | 2004 | DQ289630 |  |
| Beijing/04/44 | China | 2004 | DQ289632 |  |
| Beijing/04/54 | China | 2004 | DQ289638 |  |
| Beijing/A/04/01 | China | 2004 | DQ289630 |  |
| Beijing/A/04/09 | China | 2004 | DQ289607 |  |
| Beijing/A/04/17 | China | 2004 | DQ289612 |  |
| Beijing/A/04/22 | China | 2004 | DQ289615 |  |
| Beijing/A/04/26 | China | 2004 | DQ289613 |  |
| Beijing/A/04/37 | China | 2004 | DQ289626 |  |
| Beijing/A/04/41 | China | 2004 | DQ289629 |  |
| Beijing/A/04/43 | China | 2004 | DQ289631 |  |
| Beijing/A/04/50 | China | 2004 | DQ289635 |  |
| Beijing/A/04/51 | China | 2004 | DQ289636 |  |
| Beijing/A/04/56 | China | 2004 | DQ289639 |  |
| Lanzhou/04/01 | China | 2001 | DQ289642 |  |
| Changchun/91/01 | China | 2001 | DQ289643 |  |
| Changchun/91/04 | China | 2004 | DQ289645 |  |
| Changchun/91/05 | China | 2005 | DQ289646 |  |
| BR/A-11/04 | Brazil | 2004 | FJ804074 | [8] |
| BR/A-13/04 | Brazil | 2004 | FJ804075 |  |
| BR/A-24/04 | Brazil | 2004 | FJ804087 |  |
| BR/A-30/04 | Brazil | 2004 | FJ804076 |  |
| BR/C-13/04 | Brazil | 2004 | FJ804078 |  |
| BR/C-16/04 | Brazil | 2004 | FJ804085 |  |
| BR/C-27/04 | Brazil | 2004 | FJ804086 |  |
| BR/C-38/04 | Brazil | 2004 | FJ804079 |  |
| BR/C-4/04 | Brazil | 2004 | FJ804077 |  |
| BR/C-45/04 | Brazil | 2004 | FJ804080 |  |
| BR/C-5/04 | Brazil | 2004 | FJ804082 |  |
| BR/C-8/04 | Brazil | 2004 | FJ804088 |  |
| BR01-97 | Brazil | 1997 | EU582273 | (Botosso *et al*., unpublished) |
| BR02-02 | Brazil | 2002 | EU582056 |  |
| BR03-99 | Brazil | 1999 | EU582282 |  |
| BR04-98 | Brazil | 1998 | EU582195 |  |
| BR07-05 | Brazil | 2005 | EU582163 |  |
| BR08-96 | Brazil | 1996 | EU582192 |  |
| BR09-99 | Brazil | 1999 | EU582283 |  |
| BR10-98 | Brazil | 1998 | EU582196 |  |
| BR10-99 | Brazil | 1999 | EU582210 |  |
| BR100-99 | Brazil | 1999 | EU582298 |  |
| BR102-02 | Brazil | 2002 | EU582066 |  |
| BR102-99 | Brazil | 1999 | EU582230 |  |
| BR103-00 | Brazil | 2000 | EU582333 |  |
| BR103-03 | Brazil | 2003 | EU582125 |  |
| BR106-00 | Brazil | 2000 | EU582334 |  |
| BR106-02 | Brazil | 2002 | EU582067 |  |
| BR106-99 | Brazil | 1999 | EU582231 |  |
| BR106-99 | Brazil | 1999 | EU582231 |  |
| BR108-00 | Brazil | 2000 | EU582336 |  |
| BR108-96 | Brazil | 1996 | EU582266 |  |
| BR11-99 | Brazil | 1999 | EU582284 |  |
| BR110-00 | Brazil | 2000 | EU582337 |  |
| BR111-00 | Brazil | 2000 | EU582338 |  |
| BR112-99 | Brazil | 1999 | EU582299 |  |
| BR113-00 | Brazil | 2000 | EU582257 |  |
| BR113-96 | Brazil | 1996 | EU582268 |  |
| BR116-03 | Brazil | 2003 | EU582106 |  |
| BR117-00 | Brazil | 2000 | EU582339 |  |
| BR117-01 | Brazil | 2001 | EU582082 |  |
| BR117-96 | Brazil | 1996 | EU582169 |  |
| BR119-03 | Brazil | 2003 | EU582092 |  |
| BR12-98 | Brazil | 1998 | EU582197 |  |
| BR121-00 | Brazil | 2000 | EU582341 |  |
| BR122-00 | Brazil | 2000 | EU582342 |  |
| BR123-00 | Brazil | 2000 | EU582343 |  |
| BR124-01 | Brazil | 2001 | EU582083 |  |
| BR124-99 | Brazil | 1999 | EU582233 |  |
| BR126-99 | Brazil | 1999 | EU582300 |  |
| BR127-99 | Brazil | 1999 | EU582301 |  |
| BR128-00 | Brazil | 2000 | EU582260 |  |
| BR128-04 | Brazil | 2004 | EU582131 |  |
| BR129-00 | Brazil | 2000 | EU582261 |  |
| BR130-00 | Brazil | 2000 | EU582345 |  |
| BR131-00 | Brazil | 2000 | EU582346 |  |
| BR132-00 | Brazil | 2000 | EU582347 |  |
| BR134-00 | Brazil | 2000 | EU582348 |  |
| BR137-03 | Brazil | 2003 | EU582108 |  |
| BR139-04 | Brazil | 2004 | EU582142 |  |
| BR141-00 | Brazil | 2000 | EU582349 |  |
| BR141-03 | Brazil | 2003 | EU582113 |  |
| BR142-00 | Brazil | 2000 | EU582262 |  |
| BR142-04 | Brazil | 2004 | EU582143 |  |
| BR144-04 | Brazil | 2004 | EU582144 |  |
| BR144-96 | Brazil | 1996 | EU582269 |  |
| BR145-03 | Brazil | 2003 | EU582094 |  |
| BR147-00 | Brazil | 2000 | EU582350 |  |
| BR150-03 | Brazil | 2003 | EU582109 |  |
| BR150Del00 | Brazil | 2003 | EU582351 |  |
| BR153-00 | Brazil | 2000 | EU582350 |  |
| BR154-04 | Brazil | 2004 | EU582146 |  |
| BR155-04 | Brazil | 2004 | EU582147 |  |
| BR156-03 | Brazil | 2003 | EU582096 |  |
| BR157-00 | Brazil | 2000 | EU582353 |  |
| BR159-00 | Brazil | 2000 | EU582354 |  |
| BR161-03 | Brazil | 2003 | EU582116 |  |
| BR168-02 | Brazil | 2002 | EU582072 |  |
| BR169-04 | Brazil | 2004 | EU582133 |  |
| BR171-00 | Brazil | 2003 | EU582263 |  |
| BR171-03 | Brazil | 2003 | EU582099 |  |
| BR176-96 | Brazil | 1996 | EU582189 |  |
| BR178-04 | Brazil | 2004 | EU582149 |  |
| BR18-99 | Brazil | 1999 | EU582213 |  |
| BR186-03 | Brazil | 2003 | EU582126 |  |
| BR20-99 | Brazil | 1999 | EU582214 |  |
| BR206-04 | Brazil | 2004 | EU582150 |  |
| BR22-00 | Brazil | 2000 | EU582235 |  |
| BR22-98 | Brazil | 1998 | EU582198 |  |
| BR23-98 | Brazil | 1998 | EU582199 |  |
| BR23-99 | Brazil | 1999 | EU582216 |  |
| BR230-04 | Brazil | 2004 | EU582136 |  |
| BR233-04 | Brazil | 2004 | EU582151 |  |
| BR240-04 | Brazil | 2004 | EU582152 |  |
| BR25-99 | Brazil | 1999 | EU582217 |  |
| BR250-04 | Brazil | 2004 | EU582153 |  |
| BR251-04 | Brazil | 2004 | EU582154 |  |
| BR26-00 | Brazil | 2000 | EU582304 |  |
| BR270-04 | Brazil | 2004 | EU582157 |  |
| BR278-05 | Brazil | 2005 | EU582166 |  |
| BR28-00 | Brazil | 2000 | EU582305 |  |
| BR280-03 | Brazil | 2003 | EU582100 |  |
| BR29-98 | Brazil | 1998 | EU582200 |  |
| BR293-04 | Brazil | 2004 | EU582158 |  |
| BR301-03 | Brazil | 2003 | EU582118 |  |
| BR315-05 | Brazil | 2005 | EU582168 |  |
| BR32-98 | Brazil | 1998 | EU582201 |  |
| BR327-04 | Brazil | 2004 | EU582160 |  |
| BR34-00 | Brazil | 2000 | EU582240 |  |
| BR34-03 | Brazil | 2003 | EU582101 |  |
| BR34-98 | Brazil | 1998 | EU582277 |  |
| BR35-02 | Brazil | 2002 | EU582090 |  |
| BR37-00Del | Brazil | 2000 | EU582306 |  |
| BR37-99 | Brazil | 1999 | EU582218 |  |
| BR38-98 | Brazil | 1998 | EU582202 |  |
| BR38-99 | Brazil | 1999 | EU582219 |  |
| BR39-98 | Brazil | 1998 | EU582278 |  |
| BR43-00 | Brazil | 2000 | EU582308 |  |
| BR45-00 | Brazil | 2000 | EU582309 |  |
| BR45-98 | Brazil | 1998 | EU582279 |  |
| BR47-95 | Brazil | 1995 | EU582188 |  |
| BR48-02 | Brazil | 2002 | EU582076 |  |
| BR49-00 | Brazil | 2000 | EU582310 |  |
| BR49-96 | Brazil | 1996 | EU582190 |  |
| BR50-99 | Brazil | 1999 | EU582288 |  |
| BR51-00 | Brazil | 2000 | EU582311 |  |
| BR52-99 | Brazil | 1999 | EU582289 |  |
| BR53-00 | Brazil | 2000 | EU582246 |  |
| BR5398 | Brazil |  | EU582203 |  |
| BR54-99 | Brazil | 1999 | EU582290 |  |
| BR55-00 | Brazil | 2000 | EU582312 |  |
| BR56-00 | Brazil | 2000 | EU582247 |  |
| BR56-02 | Brazil | 2002 | EU582060 |  |
| BR56-98 | Brazil | 1998 | EU582280 |  |
| BR58-97 | Brazil | 1997 | EU582194 |  |
| BR59-01 | Brazil | 2001 | EU582055 |  |
| BR60-00 | Brazil | 2000 | EU582315 |  |
| BR60-98 | Brazil | 1998 | EU582204 |  |
| BR61-02 | Brazil | 2002 | EU582089 |  |
| BR65-00 | Brazil | 2000 | EU582316 |  |
| BR65-96 | Brazil | 1996 | EU582179 |  |
| BR65-99 | Brazil | 1999 | EU582220 |  |
| BR66-00 | Brazil | 2000 | EU582317 |  |
| BR66-03 | Brazil | 2003 | EU582120 |  |
| BR67-00 | Brazil | 2000 | EU582318 |  |
| BR67-02 | Brazil | 2002 | EU582062 |  |
| BR67-03 | Brazil | 2003 | EU582121 |  |
| BR67-97 | Brazil | 1997 | EU582186 |  |
| BR68-00 | Brazil | 2000 | EU582248 |  |
| BR68-99 | Brazil | 1999 | EU582221 |  |
| BR69-00 | Brazil | 2000 | EU582319 |  |
| BR70-00 | Brazil | 2000 | EU582320 |  |
| BR70-99 | Brazil | 1999 | EU582292 |  |
| BR71-96 | Brazil | 1996 | EU582272 |  |
| BR71Del00 | Brazil |  | EU582321 |  |
| BR72-00 | Brazil | 2000 | EU582322 |  |
| BR72-99 | Brazil | 1999 | EU582293 |  |
| BR74-95 | Brazil | 1995 | EU582265 |  |
| BR75-00 | Brazil | 2000 | EU582323 |  |
| BR75-99 | Brazil | 1999 | EU582294 |  |
| BR76-02 | Brazil | 2002 | EU582063 |  |
| BR77-99 | Brazil | 1999 | EU582295 |  |
| BR78-00 | Brazil | 2000 | EU582324 |  |
| BR79-96 | Brazil | 1996 | EU582180 |  |
| BR79-99 | Brazil | 1999 | EU582223 |  |
| BR80-99 | Brazil | 1999 | EU582224 |  |
| BR81-00 | Brazil | 2000 | EU582326 |  |
| BR81-99 | Brazil | 1999 | EU582302 |  |
| BR82-00 | Brazil | 2000 | EU582327 |  |
| BR83-02 | Brazil | 2002 | EU582085 |  |
| BR83-03 | Brazil | 2003 | EU582091 |  |
| BR84-00 | Brazil | 2000 | EU582328 |  |
| BR84-02 | Brazil | 2002 | EU582086 |  |
| BR84-03 | Brazil | 2003 | EU582104 |  |
| BR84-98 | Brazil | 1998 | EU582206 |  |
| BR85-00 | Brazil | 2000 | EU582251 |  |
| BR85-02 | Brazil | 2002 | EU582064 |  |
| BR85-99 | Brazil | 1999 | EU582226 |  |
| BR87-99 | Brazil | 1999 | EU582227 |  |
| BR88-00 | Brazil | 2000 | EU582329 |  |
| BR88-03 | Brazil | 2003 | EU582123 |  |
| BR89-00 | Brazil | 2000 | EU582253 |  |
| BR89-96 | Brazil | 1996 | EU582193 |  |
| BR89-99 | Brazil | 1999 | EU582228 |  |
| BR90-02 | Brazil | 2002 | EU582087 |  |
| BR91-03 | Brazil | 2003 | EU582105 |  |
| BR92-03 | Brazil | 2003 | EU582124 |  |
| BR94-00 | Brazil | 2000 | EU582331 |  |
| BR94-00 | Brazil | 2000 | EU582331 |  |
| BR95-00 | Brazil | 2000 | EU582255 |  |
| BR95-99 | Brazil | 1999 | EU582297 |  |
| BR97-99 | Brazil | 1999 | EU582229 |  |
| BR98-98 | Brazil | 1998 | EU582207 |  |
| BR99-02 | Brazil | 2002 | EU582088 |  |
| Brazil/1678/2007 | Brazil | 2007 | EU625718 | (Souza *et al.*, unpublished) |
| Brazil/1679/2007 | Brazil | 2007 | EU625719 |  |
| Brazil/1701/2007 | Brazil | 2007 | EU625720 |  |
| Brazil/1702/2007 | Brazil | 2007 | EU625721 |  |
| Brazil/1703/2007 | Brazil | 2007 | EU625722 |  |
| Brazil/1708/2007 | Brazil | 2007 | EU625723 |  |
| Brazil/1715/2007 | Brazil | 2007 | EU625724 |  |
| Brazil/1760/2007 | Brazil | 2007 | EU625727 |  |
| Brazil/1771/2007 | Brazil | 2007 | EU625728 |  |
| Brazil/1785/2007 | Brazil | 2007 | EU625729 |  |
| Brazil/1789/2007 | Brazil | 2007 | EU625716 |  |
| Brazil/1838/2007 | Brazil | 2007 | EU625730 |  |
| Brazil/1839/2007 | Brazil | 2007 | EU625731 |  |
| Brazil/1855/2007 | Brazil | 2007 | EU625732 |  |
| Brazil/1859/2007 | Brazil | 2007 | EU625733 |  |
| Brazil/1860/2007 | Brazil | 2007 | EU625734 |  |
| Brazil/1866/2007 | Brazil | 2007 | EU625717 |  |
| Brazil/v1742c/2007 | Brazil | 2007 | EU625726 |  |
| CH17/93 | USA | 1993 | AF065255 | [9] |
| CH57/94 | USA | 1994 | AF065258 |  |
| Chongqing/06/06 | China | 2006 | GU550444 | [10] |
| Chongqing/06/73 | China | 2006 | GU550453 |  |
| Chongqing/07/114 | China | 2007 | GU550467 |  |
| Chongqing/A/06/49 | China | 2006 | GU550452 |  |
| Chongqing/A/07/107 | China | 2007 | GU550466 |  |
| Chongqing/A/07/18 | China | 2007 | GU550469 |  |
| Chongqing/A/07/25 | China | 2007 | GU550456 |  |
| Chongqing/A/07/59 | China | 2007 | GU550461 |  |
| Chongqing/A/07/64 | China | 2007 | GU550462 |  |
| Chongqing/A/07/67 | China | 2007 | GU550464 |  |
| Chongqing/A/08/03 | China | 2008 | GU550471 |  |
| Chongqing/A/08/29 | China | 2008 | GU550473 |  |
| LZ189 | China | 2007 | GU357533 |  |
| LZ359 | China | 2007 | GU357535 |  |
| LZ418 | China | 2007 | GU357540 |  |
| LZ517 | China | 2008 | GU357541 |  |
| LZ570 | China | 2008 | GU357544 |  |
| LZY118 | China | 2009 | GU357546 |  |
| DEL/192/02/A | India | 2002 | DQ248924 | [11] |
| DEL/297/03/A | India | 2003 | DQ248926 |  |
| DEL/723/04/A | India | 2004 | DQ248930 |  |
| GER/0363/98 | Germany | 1998 | FJ391374 | [12] |
| GER/0459/01 | Germany | 2001 | FJ391394 |  |
| GER/0496/06 | Germany | 2006 | FJ391417 |  |
| GER/0503/01 | Germany | 2001 | FJ391382 |  |
| GER/0562/01 | Germany | 2001 | FJ391395 |  |
| GER/0597/02 | Germany | 2002 | FJ391402 |  |
| GER/0635/06 | Germany | 2006 | FJ391423 |  |
| GER/0702/01 | Germany | 2001 | FJ391396 |  |
| GER/0748/00 | Germany | 2000 | FJ391375 |  |
| GER/0787/07 | Germany | 2007 | FJ391435 |  |
| GER/0820/07 | Germany | 2007 | FJ391437 |  |
| GER/0825/05-06 | Germany | 2005 | FJ391414 |  |
| GER/0877/05 | Germany | 2005 | FJ391408 |  |
| GER/0939/07 | Germany | 2007 | FJ391442 |  |
| GER/0962/05 | Germany | 2005 | FJ391409 |  |
| GER/0993/07 | Germany | 2007 | FJ391438 |  |
| GER/1041/07 | Germany | 2007 | FJ391443 |  |
| GER/1049/07 | Germany | 2007 | FJ391373 |  |
| GER/1134/05 | Germany | 2005 | FJ391410 |  |
| GER/1173/04-05 | Germany | 2005 | FJ391317 |  |
| GER/1244/06 | Germany | 2006 | FJ391412 |  |
| GER/1397/07 | Germany | 2007 | FJ391429 |  |
| GER/1418/05-06 | Germany | 2005-2006 | FJ391350 |  |
| GER/1600/01 | Germany | 2001 | FJ391383 |  |
| GER/1666/05 | Germany | 2005 | FJ391411 |  |
| GER/1960/04 | Germany | 2004 | FJ391404 |  |
| GER/2166/01 | Germany | 2001 | FJ391384 |  |
| GER/2194/01 | Germany | 2001 | FJ391385 |  |
| GER/2197/04-05 | Germany | 2005 | FJ391331 |  |
| GER/2233/01 | Germany | 2001 | FJ391386 |  |
| GER/2234/00 | Germany | 2000 | FJ391378 |  |
| GER/2347/06-07 | Germany | 2007 | FJ391360 |  |
| GER/2365/04 | Germany | 2004 | FJ391405 |  |
| GER/2471/07 | Germany | 2007 | FJ391431 |  |
| GER/2498/01 | Germany | 2001 | FJ391387 |  |
| GER/2504/00 | Germany | 2000 | FJ391447 |  |
| GER/2505/00 | Germany | 2000 | FJ391379 |  |
| GER/2518/01 | Germany | 2001 | FJ391388 |  |
| GER/2526/00 | Germany | 2000 | FJ391380 |  |
| GER/2578/04 | Germany | 2004 | FJ391407 |  |
| GER/2630/01 | Germany | 2001 | FJ391390 |  |
| GER/2775/01 | Germany | 2001 | FJ391392 |  |
| GER/2946/06 | Germany | 2006 | FJ391416 |  |
| GER/3004/06-07 | Germany | 2007 | FJ391366 |  |
| GER/3694/07 | Germany | 2007 | FJ391440 |  |
| GER/3888/04-05 | Germany | 2004-2005 | FJ391336 |  |
| GER/3897/07 | Germany | 2007 | FJ391441 |  |
| INDIA/Dibrugarh/2249/09 | India | 2009 | JF907049 | (Biswas *et al*, unpublished) |
| INDIA/Dibrugarh/2269/09 | India | 2009 | JF907051 |  |
| INDIA/Dibrugarh/2272/09 | India | 2009 | JF907052 |  |
| INDIA/Dibrugarh/2899/10 | India | 2010 | JF907055 |  |
| INDIA/PN/14497/91 | India | 1991 | EU502914 | (Yeolekar *et al*., unpublished) |
| INDIA/PN/18172/06 | India | 2006 | EU267347 |  |
| INDIA/PN/18551/06 | India | 2006 | EU267352 |  |
| INDIA/PN/5256/94 | India | 1994 | EU502916 |  |
| INDIA/PN/5459/94 | India | 1995 | EU502917 |  |
| INDIA/PN/5879/95 | India | 1995 | EU502919 |  |
| INDIA/PN/18905/06 | India | 2006 | EU267346 | (Damle *et al*., unpublished) |
| INDIA/PN/18906/06 | India | 2006 | EU267345 |  |
| INDIA/PN/18971A/06 | India | 2006 | EU267343 |  |
| INDIA/PN/18981/06 | India | 2006 | EU267348 |  |
| INDIA/PN/2032/93 | India | 1993 | EU502915 |  |
| INDIA/PN/22575/06 | India | 2006 | EU267351 |  |
| INDIA/PN/6314/02 | India | 2002 | EU267368 |  |
| INDIA/PN/7081/02 | India | 2002 | EU267369 |  |
| INDIA/PN/22627/06 | India | 2006 |  |  |
| IST-A-15 | Turkey | unpublished | AY465940 | (Midilli *et al*., unpublished) |
| IST-A-20 | Turkey | unpublished | AY465943 |  |
| IST-A-22 | Turkey | unpublished | AY465944 |  |
| IST-A-5/06 | Turkey | unpublished | DQ985744 |  |
| IST-A-9 | Turkey | unpublished | AY465940 |  |
| Italy/RMa1997/98 | Italy | 1997-1998 | EU025187 | [13] |
| Italy/RMa2000/01 | Italy | 2000-2001 | EU025190 |  |
| Italy/RMa2001/02 | Italy | 2001-2002 | EU025189 |  |
| Italy/RMa2005/06 | Italy | 2005-2006 | EU025191 |  |
| Italy/RMb2002/03 | Italy | 2002-2003 | EU025192 |  |
| Italy/RMc2001/02 | Italy | 2001-2002 | EU025195 |  |
| Italy/RMc2005/06 | Italy | 2005-2006 | EU0251971 |  |
| Italy/RMd2001/02 | Italy | 2001-2002 | EU025198 |  |
| Italy/RMd2005/06 | Italy | 2005-2006 | EU025200 |  |
| Italy/RMe2002/03 | Italy | 2002-2003 | EU025201 |  |
| Italy/RMf2005/06 | Italy | 2005-2006 | EU0252031 |  |
| Italy/RMg2005/06 | Italy | 2005-2006 | EU025204 |  |
| Italy/TOa1999/00 | Italy | 1999-2000 | EU0252051 |  |
| Italy/TOa2000/01 | Italy | 2000-2001 | EU025206 |  |
| Italy/TOa2001/02 | Italy | 2001-2002 | EU025207 |  |
| Italy/TOa2002/03 | Italy | 2002-2003 | EU0252081 |  |
| Italy/TOb1999/00 | Italy | 1999-2000 | EU025216 |  |
| Italy/TOb2000/01 | Italy | 2000-2001 | EU025217 |  |
| Italy/TOb2001/02 | Italy | 2001-2002 | EU025218 |  |
| Italy/TOb2004/05 | Italy | 2004-2005 | EU0252201 |  |
| Italy/TOc1999/00 | Italy | 1999-2000 | EU025222 |  |
| Italy/TOd1999/00 | Italy | 1999-2000 | EU025227 |  |
| Italy/TOd2000/01 | Italy | 2000-2001 | EU025228 |  |
| Italy/TOd2001/02 | Italy | 2001-2002 | EU025229 |  |
| Italy/TOd2004/05 | Italy | 2004-2000 | EU0252301 |  |
| Italy/TOe1999/00 | Italy | 1999-2000 | EU025231 |  |
| Italy/TOe2000/01 | Italy | 2000-2001 | EU025232 |  |
| Italy/TOe2001/02 | Italy | 2001-2002 | EU025233 |  |
| Italy/TOg2002/03 | Italy | 2002-2003 | EU025239 |  |
| Italy/TOg2004/05 | Italy | 2004-2005 | EU0252401 |  |
| Italy/TOh2002/03 | Italy | 2002-2003 | EU025242 |  |
| Italy/TOh2004/05 | Italy | 2004-2005 | EU0252431 |  |
| Italy/TOi2002/03 | Italy | 2002-2003 | EU025244 |  |
| Italy/TOl2004/05 | Italy | 2004-2005 | EU025212 |  |
| Italy/TOn2004/05 | Italy | 2004-2005 | EU0252141 |  |
| Italy/TOa2004/05 | Italy | 2004-2005 | EU025209 |  |
| Italy/TOo2004/05 | Italy | 2004-2005 | EU025215 |  |
| Jiangxi/06/205 | China | 2006 | FJ157346 | (Xiong *et al*., unpublished) |
| Jiangxi/07/134 | China | 2007 | FJ157343 |  |
| Jiangxi/07/230 | China | 2007 | FJ157347 |  |
| Jiangxi/152/06 | China | 2006 | FJ157344 |  |
| JPN/Okinawa/230.08 | Japan | unpublished | AB485787 | [Nakamura *et al.*, 2009](#_ENREF_20)) |
| JPN/Okinawa/231.08 | Japan | unpublished | AB485788 |  |
| JPN/Okinawa/249.08 | Japan | unpublished | AB485789 |  |
| Kenya/10/01 | Kenya | 2001 | AY524581 | [14] |
| Kenya/11/02 | Kenya | 2002 | AY524584 |  |
| Kenya/123/02 | Kenya | 2002 | AY524589 |  |
| Kenya/15/01 | Kenya | 2001 | AY524598 |  |
| Kenya/158/02 | Kenya | 2002 | AY524600 |  |
| Kenya/160/02 | Kenya | 2002 | AY524604 |  |
| Kenya/164/02 | Kenya | 2002 | AY524608 |  |
| Kenya/168/02 | Kenya | 2002 | AY524610 |  |
| Kenya/196/02 | Kenya | 2002 | AY773299 |  |
| Kenya/199/02 | Kenya | 2002 | AY773296 |  |
| Kenya/232/02 | Kenya | 2002 | AY773288 |  |
| Kenya/3/00 | Kenya | 2000 | AY524635 |  |
| Kenya/4/03 | Kenya | 2003 | AY660679 |  |
| Kenya/43/02 | Kenya | 2002 | AY524641 |  |
| Kenya/5/00 |  | 2000 | AY524643 |  |
| Kenya/5/03 | Kenya | 2003 | AY660677 |  |
| Kenya/54/02 |  | 2002 | AY524646 |  |
| Kenya/8/01 | Kenya | 2001 | AY524658 |  |
| Korea//CB251/09 | South Korea | 2009 | HQ699270 | [15] |
| Korea/CB1012/10 | South Korea | 2010 | HQ699275 |  |
| Korea/CB1014/10 | South Korea | 2010 | HQ699276 |  |
| Korea/CB1051/10 | South Korea | 2010 | HQ699277 |  |
| Korea/CB132/08 | South Korea | 2008 | HQ699267 |  |
| Korea/CB153/08 | South Korea | 2008 | HQ699268 |  |
| Korea/CB22/08 | South Korea | 2008 | HQ699265 |  |
| Korea/CB339/09 | South Korea | 2009 | HQ699271 |  |
| Korea/CB767/09 | South Korea | 2009 | HQ699283 |  |
| Korea/CB824/09 | South Korea | 2009 | HQ699278 |  |
| Korea/CB836/09 | South Korea | 2009 | HQ699272 |  |
| Korea/CB851/09 | South Korea | 2009 | HQ699279 |  |
| Korea/CB904/09 | South Korea | 2009 | HQ699274 |  |
| Korea/CB931/10 | South Korea | 2010 | HQ699284 |  |
| Korea/CB932/10 | South Korea | 2010 | HQ699285 |  |
| LV/022/10 | Unpublished data | 2010 | JF979152 | (Balmaks *et al*., unpublished) |
| MAD-3-89 | Spain | 1989 | Z33415 | [16] |
| MON-1-89 | Uruguay | 1989 | Z33422 |  |
| MON-2-88 | Uruguay | 1988 | Z33424 |  |
| MON-3-88 | Uruguay | 1988 | Z33425 |  |
| Moz/12/99 | Mozambique | 1999 | AF309656 | [17] |
| Moz/170/99 | Mozambique | 1999 | AF309663 |  |
| Moz/27/99 | Mozambique | 1999 | AF309661 |  |
| Moz/33/99 | Mozambique | 1999 | AF309658 |  |
| NCL21540/97.0 | England | 1997 | HQ731699 | [18] |
| NCL24882/96.0 | England | 1996 | HQ731701 |  |
| NCL25137/96.0 | England | 1996 | HQ731703 |  |
| NCL409/09.0 | England | 2009 | HQ731705 |  |
| NCL8923/97.0 | England | 1997 | HQ731710 |  |
| NCLE8453/76.0 | England | 1976 | HQ731715 |  |
| NCLH1414/81.0 | England | 1981 | HQ731716 |  |
| NCLL2782/85.0 | England | 1985 | HQ731719 |  |
| NCLMBWS2/84.0 | England | 1984 | HQ731720 |  |
| NCLP1641/91.0 | England | 1991 | HQ731721 |  |
| NL20752227/07-08 | Netherlands | 2005 | HQ731761 |  |
| NL20752314/07-08 | Netherlands | 2005 | HQ731762 |  |
| R14466/08-09 | Scotland | 2009 | HQ731726 |  |
| R25805/09-10 | Scotland | 2010 | HQ731730 |  |
| R3318/06-07 | Scotland | 2007 | HQ731773 |  |
| R3452/06-07 | Scotland | 2007 | HQ731774 |  |
| R9088/07-08 | Scotland | 2008 | HQ731738 |  |
| UK/NCL2409_09.0/97 | England | 2009 | HQ731700 |  |
| UK/NCL3909/09 | England | 2009 | GU386537 |  |
| UK/NCL4808/08 | England | 2008 | GU386539 |  |
| UK/NCL8923/97 | England | 1996 | GU386543 |  |
| UK/NCLR17138/94.0 | England | 1994 | HQ731723 |  |
| UK/R14400/08-09 | Scotland | 2009 | HQ731728 |  |
| UK/R14414/08-09 | Scotland | 2009 | HQ731732 |  |
| UK/R14415/08-09 | Scotland | 2009 | HQ731735 |  |
| UK/R14429/08-09 | Scotland | 2009 | HQ731727 |  |
| UK/R25827/09-10 | Scotland | 2010 | HQ731733 |  |
| UK/R25993/09-10 | Scotland | 2010 | HQ731740 |  |
| Thai/BK153/06 | Thailand | 2006 | HQ731694 |  |
| BK3/06-07 | Thailand | 2006 | HQ731778 |  |
| NG-001-02 | Japan | 2005 | AB175814 | [19] |
| NG-009-02 | Japan | 2005 | AB175815 |  |
| NG-040-03 | Japan | 2005 | AB175818 |  |
| NG-100-08 | Japan | 2005 | AB603446 |  |
| NG/001/03 | Japan | 2003 | AB603461 |  |
| NG/004/04 | Japan | 2004 | AB603459 |  |
| NG/058/04 | Japan | 2004 | AB603458 |  |
| NG/069/05 | Japan | 2005 | AB603447 |  |
| NG/114/03 | Japan | 2005 | AB603465 |  |
| NZA_89_01 | New Zealand | 1989 | DQ171762 | [20] |
| ON97-0111A | Canada | 2011 | JN257703 | [21] |
| RP019/06 | Brazil | 2006 | EU635829 | (Proenca-Modena *et al*., unpublished) |
| RP035/06 | Brazil | 2006 | EU635831 |  |
| RP040/06 | Brazil | 2006 | EU635832 |  |
| RP046/06 | Brazil | 2006 | EU635833 |  |
| RP047/06 | Brazil | 2006 | EU635834 |  |
| RP050/06 | Brazil | 2006 | EU635835 |  |
| RP065/06 | Brazil | 2006 | EU635836 |  |
| RP066/06 | Brazil | 2006 | EU635837 |  |
| RP079/05 | Brazil | 2005 | EU635817 |  |
| RP092/04 | Brazil | 2004 | EU635778 |  |
| RP099/04 | Brazil | 2004 | EU635780 |  |
| RP106/04 | Brazil | 2004 | EU635781 |  |
| RP109/05 | Brazil | 2005 | EU635819 |  |
| RP110/04 | Brazil | 2004 | EU635783 |  |
| RP116/04 | Brazil | 2004 | EU635784 |  |
| RP119/05 | Brazil | 2005 | EU635821 |  |
| RP120/04 | Brazil | 2004 | EU635785 |  |
| RP124/04 | Brazil | 2004 | EU635786 |  |
| RP125/06 | Brazil | 2005 | EU635838 |  |
| RP126/06 | Brazil | 2006 | EU635839 |  |
| RP127/04 | Brazil | 2004 | EU635788 |  |
| RP129/04 | Brazil | 2004 | EU635790 |  |
| RP130/05 | Brazil | 2005 | EU635822 |  |
| RP131/04 | Brazil | 2004 | EU635791 |  |
| RP133/04 | Brazil | 2004 | EU635792 |  |
| RP136/04 | Brazil | 2004 | EU635793 |  |
| RP138/04 | Brazil | 2004 | EU635794 |  |
| RP139/04 | Brazil | 2004 | EU635802 |  |
| RP145/04 | Brazil | 2004 | EU635796 |  |
| RP150/04 | Brazil | 2004 | EU635797 |  |
| RP155/06 | Brazil | 2006 | EU635840 |  |
| RP163/04 | Brazil | 2004 | EU635799 |  |
| RP170/04 | Brazil | 2004 | EU635800 |  |
| RP175/04 | Brazil | 2004 | EU635802 |  |
| RP176/05 | Brazil | 2005 | EU635826 |  |
| RP181/04 | Brazil | 2004 | EU635803 |  |
| RP186/06 | Brazil | 2006 | EU635841 |  |
| RP187/06 | Brazil | 2006 | EU635842 |  |
| RP190/04 | Brazil | 2004 | EU635804 |  |
| RP191/06 | Brazil | 2006 | EU635844 |  |
| RP193/04 | Brazil | 2004 | EU635805 |  |
| RP200/04 | Brazil | 2004 | EU635806 |  |
| RP201/04 | Brazil | 2004 | EU635807 |  |
| RP205/06 | Brazil | 2006 | EU635846 |  |
| RP209/04 | Brazil | 2004 | EU635810 |  |
| RP210/06 | Brazil | 2006 | EU635848 |  |
| RP211/04 | Brazil | 2004 | EU635811 |  |
| RP212/06 | Brazil | 2006 | EU635849 |  |
| RP214/04 | Brazil | 2004 | EU635812 |  |
| RP218/04 | Brazil | 2004 | EU635813 |  |
| RP219/04 | Brazil | 2004 | EU635814 |  |
| RP239/04 | Brazil | 2004 | EU635816 |  |
| SA/0069KS/01 | South Africa | 2001 | AY226511 | [22] |
| SA/0128KS/01 | South Africa | 2001 | AY226512 |  |
| SA/1491542/08 | South Africa | 2008 |  |  |
| SA/G148S/01 | South Africa | 2001 | AY226516 |  |
| SA/G160S/01 | South Africa | 2001 | AY226517 |  |
| SA/G58S/01 | South Africa | 2001 | AY226518 |  |
| SA/157KS/01 | South Africa | 2001 | AY226515 |  |
| SA/Ab11B00 | South Africa | | AY146422 | [23] |
| SA/Ab20Bl00 | South Africa | | AY146415 |  |
| SA/Ab21Bl00 | South Africa | | AY146420 |  |
| SA/Ab24Ct00 | South Africa | | AY146417 |  |
| SA/Ab3059CT01 | South Africa | | AY146433 |  |
| SA/Ab3061CT01 | South Africa | | AY146434 |  |
| SA/Ab31Ct00 | South Africa | | AY146421 |  |
| SA/Ab4026B01 | South Africa | | AY146435 |  |
| SA/Ab4029B/01 | South Africa | | AY146436 |  |
| SA/Ab43Ct00 | South Africa | | AY146418 |  |
| SA/Ab47Pt00 | South Africa | | AY146429 |  |
| SA/Ab5076Pt/01 | South Africa | | AY146437 |  |
| SA/Ab54Pt00 | South Africa | | AY146419 |  |
| SA/Ab55Pt00 | South Africa | | AY146424 |  |
| SA/Ab71D00 | South Africa | | AY146412 |  |
| SA/Ab84J00 | South Africa | | AY146427 |  |
| SA/Ab86J00 | South Africa | | AY146428 |  |
| SA/Ag_A48-99 | South Africa | | AF548801 | [24] |
| SA/Ag32-00 | South Africa | | AF548802 |  |
| SA/Ag38-00 | South Africa | | AF548797 |  |
| SA/AgK23-00 | South Africa | | AF548799 |  |
| SA/AgK28-00 | South Africa | | AF548798 |  |
| SA/D1289/97 | South Africa | | AF348803 | [25] |
| SA/D804/97 | South Africa | | AF348802 |  |
| SA/V360/99 | South Africa | | AF348804 |  |
| SA97D669 | South Africa | 1997 | AF348809 |  |
| SA97D804 | South Africa | 1997 | AF348802 |  |
| SA98D707 | South Africa | 1998 | AF348810 |  |
| SA98V603 | South Africa | 1998 | AF348807 |  |
| SA99V1239 | South Africa | 1999 | AF348808 |  |
| SA0003 | South Africa | | AF348805 |  |
| SA394753K06 | South Africa | 2006 | EF212032 | [26] |
| SA445197K06 | South Africa | 2006 | EF212033 |  |
| SA452962K06 | South Africa | 2008 | EF212035 |  |
| SAL/108/99 | Brazil | 1999 | AY472096 | [27] |
| SAL/136/99 | Brazil | 1999 | AY472089 |  |
| SAL/140/99 | Brazil | 1999 | AY472087 |  |
| SAL/149/99 | Brazil | 1999 | AY472088 |  |
| SAL/151/99 | Brazil | 1999 | AY472090 |  |
| SAL/154/99 | Brazil | 1999 | AY472091 |  |
| SAL/173/99 | Brazil | 1999 | AY472094 |  |
| SAL/81/99 | Brazil | 1999 | AY472095 |  |
| SAL/87/99 | Brazil | 1999 | AY472086 |  |
| SEL/97385/97 | South Korea | 1997 | AF193323 | [28] |
| SEL/97434/97 | South Korea | 1997 | AF193324 |  |
| SEL/98072/98 | South Korea | 1998 | AF193325 |  |
| Singapore/LLC62-111/00-01 | Singapore | 2000-2001 | AY114151 | [29] |
| SP1401/2006 | Brazil | 2006 | EU625712 | (Souza *et al*., unpublished) |
| Tehran/A/08/788 | Iran | 2008 | GU339401 | [30] |
| V00-2221902 | Hong Kong | 2000 | JN968312 | (Mak *et al.,* unpublished) |
| V00-2225288 | Hong Kong | 2000 | JN968333 |  |
| V01-2203685 | Hong Kong | 2001 | JN968334 |  |
| V01-2209978 | Hong Kong | 2001 | JN968335 |  |
| V01-2222398 | Hong Kong | 2001 | JN968336 |  |
| V02-2203034 | Hong Kong | 2002 | JN968337 |  |
| V02-2208039 | Hong Kong | 2002 | JN968338 |  |
| V02-2215330 | Hong Kong | 2002 | JN968339 |  |
| V02-2219452 | Hong Kong | 2002 | JN968314 |  |
| V02-2224573 | Hong Kong | 2002 | JN968340 |  |
| V04-2205427 | Hong Kong | 2004 | JN968341 |  |
| V05-2246573 | Hong Kong | 2005 | JN968315 |  |
| V06-2215072 | Hong Kong | 2006 | JN968316 |  |
| V06-2224920 | Hong Kong | 2006 | JN968332 |  |
| V06-2240346 | Hong Kong | 2006 | JN968317 |  |
| V06-2262605 | Hong Kong | 2006 | JN968318 |  |
| V07-2207622 | Hong Kong | 2007 | JN968319 |  |
| V07-2221632 | Hong Kong | 2007 | JN968320 |  |
| V07-2229208 | Hong Kong | 2007 | JN968298\| |  |
| V07-2246878 | Hong Kong | 2007 | JN968321 |  |
| V07-2257560 | Hong Kong | 2007 | JN968322 |  |
| V08-2206709 | Hong Kong | 2008 | JN968325 |  |
| V08-2220141 | Hong Kong | 2008 | JN968323 |  |
| V08-2242908 | Hong Kong | 2008 | JN968324 |  |
| V09-2206560 | Hong Kong | 2009 | JN968313 |  |
| V10-2281116 | Hong Kong | 2010 | JN968327 |  |
| V10-2282947 | Hong Kong | 2010 | JN968300 |  |
| V10-2293186 | Hong Kong | 2010 | JN968328 |  |
| V10-2293739 | Hong Kong | 2010 | JN968302 |  |
| V11-2200505 | Hong Kong | 2011 | JN968331 |  |
| V11-2214216 | Hong Kong | 2011 | JN968329 |  |
| V11-2224251 | Hong Kong | 2011 | JN968330 |  |
| V11-2235695 | Hong Kong | 2011 | JN968304 |  |
| V11-2235714 | Hong Kong | 2011 | JN968305 |  |
| V11-2235823 | Hong Kong | 2011 | JN968306 |  |
| V11-2236154 | Hong Kong | 2011 | JN968307 |  |
| V11-2236212 | Hong Kong | 2011 | JN968308 |  |
| V11-2236213 | Hong Kong | 2011 | JN968309 |  |
| V11-2236216 | Hong Kong | 2011 | JN968310 |  |
| V11-2236286 | Hong Kong | 2011 | JN968311 |  |
| V11-2241152 | Hong Kong | 2011 | JN968326 |  |
| WV/12342/84 | USA | 1984 | AF065408 | [31] |
| WV/23836/88 | USA | 1988 | AF065410 |  |
| WV/5222/81 | USA | 1981 | AF065406 |  |
| Zhejiang/04/001 | China |  | AY728167 | (Mao *et al*., unpublished) |

1. Anderson LJ, Hierholzer JC, Tsou C, Hendry RM, Fernie BF, et al. (1985) Antigenic characterization of respiratory syncytial virus strains with monoclonal antibodies. J Infect Dis 151: 626-633.

2. Rebuffo-Scheer C, Bose M, He J, Khaja S, Ulatowski M, et al. (2011) Whole genome sequencing and evolutionary analysis of human respiratory syncytial virus A and B from Milwaukee, WI 1998-2010. PLoS One 6: e25468.

3. Peret TC, Hall CB, Hammond GW, Piedra PA, Storch GA, et al. (2000) Circulation patterns of group A and B human respiratory syncytial virus genotypes in 5 communities in North America. J Infect Dis 181: 1891-1896.

4. Frabasile S, Delfraro A, Facal L, Videla C, Galiano M, et al. (2003) Antigenic and genetic variability of human respiratory syncytial viruses (group A) isolated in Uruguay and Argentina: 1993-2001. J Med Virol 71: 305-312.

5. Galiano MC, Palomo C, Videla CM, Arbiza J, Melero JA, et al. (2005) Genetic and antigenic variability of human respiratory syncytial virus (groups a and b) isolated over seven consecutive seasons in Argentina (1995 to 2001). Journal of Clinical Microbiology 43: 2266-2273.

6. Zlateva KT, Lemey P, Vandamme AM, Van Ranst M (2004) Molecular evolution and circulation patterns of human respiratory syncytial virus subgroup a: positively selected sites in the attachment g glycoprotein. J Virol 78: 4675-4683.

7. Zhang Y, Xu W, Shen K, Xie Z, Sun L, et al. (2007) Genetic variability of group A and B human respiratory syncytial viruses isolated from 3 provinces in China. Arch Virol 152: 1425-1434.

8. da Silva LH, Spilki FR, Riccetto AG, de Almeida RS, Baracat EC, et al. (2008) Genetic variability in the G protein gene of human respiratory syncytial virus isolated from the Campinas metropolitan region, Brazil. J Med Virol 80: 1653-1660.

9. Peret TC, Hall CB, Schnabel KC, Golub JA, Anderson LJ (1998) Circulation patterns of genetically distinct group A and B strains of human respiratory syncytial virus in a community. J Gen Virol 79 ( Pt 9): 2221-2229.

10. Zhang RF, Jin Y, Xie ZP, Liu N, Yan KL, et al. (2010) Human respiratory syncytial virus in children with acute respiratory tract infections in China. Journal of Clinical Microbiology 48: 4193-4199.

11. Parveen S, Sullender WM, Fowler K, Lefkowitz EJ, Kapoor SK, et al. (2006) Genetic variability in the G protein gene of group A and B respiratory syncytial viruses from India. Journal of Clinical Microbiology 44: 3055-3064.

12. Reiche J, Schweiger B (2009) Genetic variability of group A human respiratory syncytial virus strains circulating in Germany from 1998 to 2007. Journal of Clinical Microbiology 47: 1800-1810.

13. Montieri S, Puzelli S, Ciccozzi M, Calzoletti L, Di Martino A, et al. (2007) Amino acid changes in the attachment G glycoprotein of human respiratory syncytial viruses (subgroup A) isolated in Italy over several epidemics (1997-2006). J Med Virol 79: 1935-1942.

14. Scott PD, Ochola R, Ngama M, Okiro EA, Nokes DJ, et al. (2004) Molecular epidemiology of respiratory syncytial virus in Kilifi district, Kenya. J Med Virol 74: 344-354.

15. Baek YH, Choi EH, Song MS, Pascua PN, Kwon HI, et al. (2012) Prevalence and genetic characterization of respiratory syncytial virus (RSV) in hospitalized children in Korea. Arch Virol 157: 1039-1050.

16. Garcia O, Martin M, Dopazo J, Arbiza J, Frabasile S, et al. (1994) Evolutionary pattern of human respiratory syncytial virus (subgroup A): cocirculating lineages and correlation of genetic and antigenic changes in the G glycoprotein. J Virol 68: 5448-5459.

17. Roca A, Loscertales MP, Quinto L, Perez-Brena P, Vaz N, et al. (2001) Genetic variability among group A and B respiratory syncytial viruses in Mozambique: identification of a new cluster of group B isolates. J Gen Virol 82: 103-111.

18. Gaunt ER, Jansen RR, Poovorawan Y, Templeton KE, Toms GL, et al. (2011) Molecular epidemiology and evolution of human respiratory syncytial virus and human metapneumovirus. PLoS One 6: e17427.

19. Sato M, Saito R, Sakai T, Sano Y, Nishikawa M, et al. (2005) Molecular epidemiology of respiratory syncytial virus infections among children with acute respiratory symptoms in a community over three seasons. Journal of Clinical Microbiology 43: 36-40.

20. Matheson JW, Rich FJ, Cohet C, Grimwood K, Huang QS, et al. (2006) Distinct patterns of evolution between respiratory syncytial virus subgroups A and B from New Zealand isolates collected over thirty-seven years. J Med Virol 78: 1354-1364.

21. Eshaghi A, Duvvuri VR, Lai R, Nadarajah JT, Li A, et al. (2012) Genetic variability of human respiratory syncytial virus a strains circulating in ontario: a novel genotype with a 72 nucleotide G gene duplication. PLoS One 7: e32807.

22. Agenbach E, Tiemessen CT, Venter M (2005) Amino acid variation within the fusion protein of respiratory syncytial virus subtype A and B strains during annual epidemics in South Africa. Virus Genes 30: 267-278.

23. Madhi SA, Ludewick H, Abed Y, Klugman KP, Boivin G (2003) Human metapneumovirus-associated lower respiratory tract infections among hospitalized human immunodeficiency virus type 1 (HIV-1)-infected and HIV-1-uninfected African infants. Clin Infect Dis 37: 1705-1710.

24. Venter M, Collinson M, Schoub BD (2002) Molecular epidemiological analysis of community circulating respiratory syncytial virus in rural South Africa: Comparison of viruses and genotypes responsible for different disease manifestations. J Med Virol 68: 452-461.

25. Venter M, Madhi SA, Tiemessen CT, Schoub BD (2001) Genetic diversity and molecular epidemiology of respiratory syncytial virus over four consecutive seasons in South Africa: identification of new subgroup A and B genotypes. J Gen Virol 82: 2117-2124.

26. Visser A, Delport S, Venter M (2008) Molecular epidemiological analysis of a nosocomial outbreak of respiratory syncytial virus associated pneumonia in a kangaroo mother care unit in South Africa. J Med Virol 80: 724-732.

27. Moura FE, Blanc A, Frabasile S, Delfraro A, de Sierra MJ, et al. (2004) Genetic diversity of respiratory syncytial virus isolated during an epidemic period from children of northeastern Brazil. J Med Virol 74: 156-160.

28. Choi EH, Lee HJ (2000) Genetic diversity and molecular epidemiology of the G protein of subgroups A and B of respiratory syncytial viruses isolated over 9 consecutive epidemics in Korea. J Infect Dis 181: 1547-1556.

29. Lim CS, Kumarasinghe G, Chow VT (2003) Sequence and phylogenetic analysis of SH, G, and F genes and proteins of Human respiratory syncytial virus isolates from Singapore. Acta Virol 47: 97-104.

30. Faghihloo E, Rezaie F, Salimi V, Naseri M, Mamishi S, et al. (2011) Molecular epidemiology of human respiratory syncytial virus in Iran. Acta Virol 55: 81-83.

31. Sullender WM, Mufson MA, Prince GA, Anderson LJ, Wertz GW (1998) Antigenic and genetic diversity among the attachment proteins of group A respiratory syncytial viruses that have caused repeat infections in children. J Infect Dis 178: 925-932.
