# Supplementary material for: Circulation of HRSV in Belgium: From Multiple Genotype Circulation to Prolonged Circulation of Predominant Genotypes
Source: PLoS One. 2013 Apr 5;8(4):e60416. doi: 10.1371/journal.pone.0060416 (PMC3618235; doi:10.1371/journal.pone.0060416)
Supplement: Table S2 — HRSV-B sequence data used in the coalescent analysis. (DOCX) [file pone.0060416.s004.docx]

**Table S2. HRSV-B sequence data used in the coalescent analysis**

| **RSV-B strain** | **Country** | **Isolation year** | **Accession Number** | **Reference** |
| --- | --- | --- | --- | --- |
| Sap/4/00 | Japan | 2005 | AB117522 | [1] |
| FS/323/2007 | Japan | 2007 | AB687662 | (Nakamura *et al.,* unpublished) |
| FS/330/2007 | Japan | 2007 | AB687664 |  |
| FS/359/2007 | Japan | 2007 | AB687666 |  |
| FS/369/2007 | Japan | 2007 | AB687668 |  |
| FS/384/2007 | Japan | 2007 | AB687670 |  |
| FS/400/2007 | Japan | 2007 | AB687672 |  |
| FS/409/2007 | Japan | 2007 | AB687674 |  |
| FS/411/2007(B) | Japan | 2007 | AB687676 |  |
| FS/421/2007 | Japan | 2007 | AB687678 |  |
| FS/440/2007 | Japan | 2007 | AB687680 |  |
| BA/3833/99 | Argentina | 1999 | AY333362 | [2] |
| BA/3859/99 | Argentina | 1999 | AY333363 |  |
| BE/12358/02 | Belgium | 2002 | AY751084 | [3] |
| BE/12670/01 | Belgium | 2001 | AY751086 |  |
| BE/13457/03 | Belgium | 2003 | AY751087 |  |
| BE/12973/03 | Belgium | 2003 | AY751089 |  |
| BE/12817/03 | Belgium | 2003 | AY751091 |  |
| BE/14610/03 | Belgium | 2003 | AY751093 |  |
| BE/12445/99 | Belgium | 1999 | AY751094 |  |
| BE/11609/01 | Belgium | 2001 | AY751102 |  |
| BE/1162/02 | Belgium | 2002 | AY751104 |  |
| BE/11500/01 | Belgium | 2001 | AY751105 |  |
| BE/12595/01 | Belgium | 2001 | AY751107 |  |
| BE/12598/01 | Belgium | 2001 | AY751108 |  |
| BE/1802/03 | Belgium | 2003 | AY751109 |  |
| BE/210/03 | Belgium | 2003 | AY751111 |  |
| BE/11508/01 | Belgium | 2001 | AY751116 |  |
| BE/13159/02 | Belgium | 2002 | AY751117 |  |
| BE/12370/01 | Belgium | 2001 | AY751118 |  |
| BE/12522/01 | Belgium | 2001 | AY751119 |  |
| BE/12349/02 | Belgium | 2002 | AY751121 |  |
| BE/1066/03 | Belgium | 2003 | AY751123 |  |
| BE/302/04 | Belgium | 2004 | AY751124 |  |
| BE/12446/01 | Belgium | 2001 | AY751125 |  |
| BE/11535/01 | Belgium | 2001 | AY751126 |  |
| BE/13417/99 | Belgium | 1999 | AY751131 |  |
| NZB_04_02 | New Zealand | 2004 | DQ171878 | [4] |
| BA/802/99 | Argentina | 1999 | DQ2273631 | [2] |
| BA/1370/99 | Argentina | 1999 | DQ227364 |  |
| BA/3931/99 | Argentina | 1999 | DQ2273651 |  |
| BA/3997/99 | Argentina | 1999 | DQ227366 |  |
| BA/164/02 | Argentina | 2002 | DQ2273671 |  |
| BA/166/02 | Argentina | 2002 | DQ227368 |  |
| BA/167/02 | Argentina | 2002 | DQ227369 |  |
| BA/495/02 | Argentina | 2002 | DQ2273701 |  |
| BA/505/02 | Argentina | 2002 | DQ2273711 |  |
| BA/619/02 | Argentina | 2002 | DQ2273721 |  |
| BA/770/02 | Argentina | 2002 | DQ2273731 |  |
| BA/733/02 | Argentina | 2002 | DQ227374 |  |
| BA/998/02 | Argentina | 2002 | DQ227375 |  |
| BA/1004/02 | Argentina | 2002 | DQ2273761 |  |
| BA/1208/02 | Argentina | 2002 | DQ2273781 |  |
| BA/1214/02 | Argentina | 2002 | DQ2273791 |  |
| BA/1271/02 | Argentina | 2002 | DQ227380 |  |
| BA/1441/02 | Argentina | 2002 | DQ2273811 |  |
| BA/1461/02 | Argentina | 2002 | DQ227382 |  |
| BA/1518/02 | Argentina | 2002 | DQ227383 |  |
| BA/1562/02 | Argentina | 2002 | DQ227384 |  |
| BA/1565/02 | Argentina | 2002 | DQ227385 |  |
| BA/1606/02 | Argentina | 2002 | DQ2273861 |  |
| BA/1856/02 | Argentina | 2002 | DQ2273871 |  |
| BA/1889/02 | Argentina | 2002 | DQ227388 |  |
| BA/4826/03 | Argentina | 2003 | DQ227389 |  |
| BA/4915/03 | Argentina | 2003 | DQ227390 |  |
| BA/4862/03 | Argentina | 2003 | DQ227391 |  |
| BA/4974/03 | Argentina | 2003 | DQ227392 |  |
| BA/5140/03 | Argentina | 2003 | DQ227393 |  |
| BA/6564/03 | Argentina | 2003 | DQ2273941 |  |
| BA/100/04 | Argentina | 2004 | DQ227395 |  |
| BA/524/04 | Argentina | 2004 | DQ227396 |  |
| BA/1607/04 | Argentina | 2004 | DQ227397 |  |
| BA/1445/02 | Argentina | 2002 | DQ227399 |  |
| BA/693/03 | Argentina | 2003 | DQ227400 |  |
| BA/4825/03 | Argentina | 2003 | DQ227401 |  |
| BA/4830/03 | Argentina | 2003 | DQ227402 |  |
| BA/4852/03 | Argentina | 2003 | DQ227403 |  |
| BA/4909/03 | Argentina | 2003 | DQ227404 |  |
| BA/5021/03 | Argentina | 2003 | DQ227405 |  |
| BA/354/04 | Argentina | 2004 | DQ227406 |  |
| BA/493/04 | Argentina | 2004 | DQ227407 |  |
| BA/1526/04 | Argentina | 2004 | DQ227408 |  |
| DEL/39OS/04/B | India | 2004 | DQ248931 | [5] |
| DEL/1106/04/B | India | 2004 | DQ248932 |  |
| DEL/1126/04/B | India | 2004 | DQ248933 |  |
| DEL/12W/04/B | India | 2004 | DQ248934 |  |
| DEL/14W/04/B | India | 2004 | DQ248935 |  |
| DEL/1137/04/B | India | 2004 | DQ248936 |  |
| DEL/1100/04/B | India | 2004 | DQ248937 |  |
| DEL/16W/04/B | India | 2004 | DQ248938 |  |
| DEL/575/03/B | India | 2003 | DQ248939 |  |
| DEL/65/02/B | India | 2002 | DQ248940 |  |
| DEL/609/03/B | India | 2003 | DQ248941 |  |
| BE/765/05 | Belgium | 2005 | DQ985136 |  |
| BE/1787/05 | Belgium | 2005 | DQ985137 |  |
| BE/12546/04 | Belgium | 2004 | DQ985138 |  |
| BE/13127/04 | Belgium | 2004 | DQ985139 |  |
| BE/2988/05 | Belgium | 2005 | DQ985140 |  |
| BE/19/05 | Belgium | 2005 | DQ985141 |  |
| BE/12963/03 | Belgium | 2003 | DQ985142 |  |
| BE/9388/05 | Belgium | 2005 | DQ985143 |  |
| BE/10451/05 | Belgium | 2005 | DQ985144 |  |
| BE/13183/04 | Belgium | 2004 | DQ985145 |  |
| BE/9382/05 | Belgium | 2005 | DQ985146 |  |
| BE/13588/04 | Belgium | 2004 | DQ985147 |  |
| BE/13588/04 | Belgium | 2004 | DQ985147 |  |
| BE/1266/05 | Belgium | 2005 | DQ985148 |  |
| BE/1515/05 | Belgium | 2005 | DQ985149 |  |
| BE/13689/04 | Belgium | 2004 | DQ985150 |  |
| BE/13689/04 | Belgium | 2004 | DQ985150 |  |
| BE/3891/05 | Belgium | 2005 | DQ985151 |  |
| BE/13547/04 | Belgium | 2004 | DQ985152 |  |
| BE/13146/04 | Belgium | 2004 | DQ985153 |  |
| BE/13804/03 | Belgium | 2003 | DQ985154 |  |
| SA490866K06 | South Africa | 2006 | EF219430 | [6] |
| SA481582K06 | South Africa | 2006 | EF219431 |  |
| SA415319K06 | South Africa | 2006 | EF219432 |  |
| SA433941K06 | South Africa | 2006 | EF219433 |  |
| ITA/183/05 | Brazil | 2005 | EU259652 | (Souza *et al.,* unpublished) |
| ITA/186/05 | Brazil | 2005 | EU259653 |  |
| ITA/188/05 | Brazil | 2005 | EU259654 |  |
| ITA/190/05 | Brazil | 2005 | EU259655 |  |
| ITA/217/05 | Brazil | 2005 | EU259656 |  |
| ITA/221/05 | Brazil | 2005 | EU259657 |  |
| ITA/298/05 | Brazil | 2005 | EU259658 |  |
| ITA/300/05 | Brazil | 2005 | EU259659 |  |
| ITA/339/05 | Brazil | 2005 | EU259660 |  |
| JU/248/05 | Brazil | 2005 | EU259661 |  |
| JU/294/05 | Brazil | 2005 | EU259662 |  |
| SP/340/05 | Brazil | 2005 | EU259663 |  |
| JU/311/05 | Brazil | 2005 | EU259664 |  |
| JU/317/05 | Brazil | 2005 | EU259665 |  |
| JU/374/05 | Brazil | 2005 | EU259666 |  |
| SP/039/05 | Brazil | 2005 | EU259667 |  |
| SP/055/05 | Brazil | 2005 | EU259668 |  |
| JU/516/05 | Brazil | 2005 | EU259669 |  |
| SP/067/05 | Brazil | 2005 | EU259670 |  |
| SP/091/05 | Brazil | 2005 | EU259671 |  |
| SP/092/05 | Brazil | 2005 | EU259672 |  |
| SP/099/05 | Brazil | 2005 | EU259673 |  |
| SP/100/05 | Brazil | 2005 | EU259674 |  |
| SP/089/05 | Brazil | 2005 | EU259675 |  |
| SP/113/05 | Brazil | 2005 | EU259676 |  |
| SP/181/05 | Brazil | 2005 | EU259678 |  |
| SP/182/05 | Brazil | 2005 | EU259679 |  |
| SP/192/05 | Brazil | 2005 | EU259680 |  |
| SP/207/05 | Brazil | 2005 | EU259681 |  |
| SP/208/05 | Brazil | 2005 | EU259682 |  |
| SP/074/05 | Brazil | 2005 | EU259683 |  |
| SP/223/05 | Brazil | 2005 | EU259684 |  |
| SP/224/05 | Brazil | 2005 | EU259685 |  |
| SP/225/05 | Brazil | 2005 | EU259686 |  |
| SP/227/05 | Brazil | 2005 | EU259687 |  |
| SP/230/05 | Brazil | 2005 | EU259688 |  |
| SP/245/05 | Brazil | 2005 | EU259689 |  |
| SP/275/05 | Brazil | 2005 | EU259690 |  |
| SP/286/05 | Brazil | 2005 | EU259691 |  |
| SP/308/05 | Brazil | 2005 | EU259692 |  |
| SP/327/05 | Brazil | 2005 | EU259693 |  |
| SP/331/05 | Brazil | 2005 | EU259694 |  |
| SP/368/05 | Brazil | 2005 | EU259695 |  |
| SP/341/05 | Brazil | 2005 | EU259696 |  |
| SP/387/05 | Brazil | 2005 | EU259697 |  |
| SP/417/05 | Brazil | 2005 | EU259698 |  |
| SP/418/05 | Brazil | 2005 | EU259699 |  |
| SP/423/05 | Brazil | 2005 | EU259700 |  |
| SP/424/05 | Brazil | 2005 | EU259701 |  |
| SP/426/05 | Brazil | 2005 | EU259702 |  |
| SP/434/05 | Brazil | 2005 | EU259703 |  |
| SP/437/05 | Brazil | 2005 | EU259704 |  |
| SP/642/05 | Brazil | 2005 | EU259705 |  |
| SP/500/05 | Brazil | 2005 | EU259706 |  |
| JU/335/05 | Brazil | 2005 | EU259707 |  |
| JU/261/05 | Brazil | 2005 | EU259708 |  |
| JU/260/05 | Brazil | 2005 | EU363290 |  |
| SP/376/05 | Brazil | 2005 | EU363294 |  |
| DEL/ADG/05 | India | 2005 | EU368623 | (Bharaj *et al*., unpublished) |
| DEL/AEG/05 | India | 2005 | EU368624 |  |
| DEL/AFE/05 | India | 2005 | EU368625 |  |
| DEL/AGG/05 | India | 2005 | EU368626 |  |
| DEL/AID/05 | India | 2005 | EU368627 |  |
| DEL/BOO/05 | India | 2005 | EU368628 |  |
| DEL/BOD/05 | India | 2005 | EU368629 |  |
| DEL/BBA/05 | India | 2005 | EU368630 |  |
| DEL/EFW/05 | India | 2005 | EU368631 |  |
| DEL/FBW/05 | India | 2005 | EU368632 |  |
| DEL/FDW/05 | India | 2005 | EU368633 |  |
| DEL/GAW/05 | India | 2005 | EU368634 |  |
| DEL/HFW/05 | India | 2005 | EU368635 |  |
| DEL/DBE/06 | India | 2006 | EU368636 |  |
| DEL/DBG/06 | India | 2006 | EU368637 |  |
| DEL/DCO/06 | India | 2006 | EU368638 |  |
| DEL/DCD/06 | India | 2006 | EU368639 |  |
| DEL/ADI/05 | India | 2005 | EU368640 |  |
| DEL/DCG/06 | India | 2006 | EU368641 |  |
| DEL/AOGC/07 | India | 2007 | EU368642 |  |
| DEL/AFF/05 | India | 2005 | EU368643 |  |
| DEL/BBE/05 | India | 2005 | EU368644 |  |
| DEL/AIA/05 | India | 2005 | EU368645 |  |
| DEL/BOG/05 | India | 2005 | EU368646 |  |
| DEL/BAA/05 | India | 2005 | EU368647 |  |
| DEL/BEO/05 | India | 2005 | EU368648 |  |
| DEL/COF/06 | India | 2006 | EU368649 |  |
| DEL/AAOW/06 | India | 2006 | EU368650 |  |
| BR19-2001 | Brazil | 2001 | EU582432 | Botosso *et al.*, unpublished |
| BR42-2001 | Brazil | 2001 | EU582433 |  |
| BR140-2003 | Brazil | 2003 | EU582447 |  |
| BR105-2003 | Brazil | 2003 | EU582448 |  |
| BR093-2004 | Brazil | 2004 | EU582449 |  |
| BR149-2005 | Brazil | 2005 | EU582458 |  |
| BR158-2005 | Brazil | 2005 | EU582460 |  |
| BR178-2005 | Brazil | 2005 | EU582461 |  |
| BR200-2005 | Brazil | 2005 | EU582462 |  |
| BR205-2005 | Brazil | 2005 | EU5824631 |  |
| BR217-2005 | Brazil | 2005 | EU582464 |  |
| BR219-2005 | Brazil | 2005 | EU582465 |  |
| BR179-2005 | Brazil | 2005 | EU582466 |  |
| BR216-2005 | Brazil | 2005 | EU582467 |  |
| BR222-2005 | Brazil | 2005 | EU582468 |  |
| BR233-2005 | Brazil | 2005 | EU582469 |  |
| BR236-2005 | Brazil | 2005 | EU582470 |  |
| BR237-2005 | Brazil | 2005 | EU582471 |  |
| BR242-2005 | Brazil | 2005 | EU582472 |  |
| BR252-2005 | Brazil | 2005 | EU582473 |  |
| BR259-2005 | Brazil | 2005 | EU582474 |  |
| BR271-2005 | Brazil | 2005 | EU582475 |  |
| BR274-2005 | Brazil | 2005 | EU582476 |  |
| BR290-2005 | Brazil | 2005 | EU582477 |  |
| BR293-2005 | Brazil | 2005 | EU582478 |  |
| BR301-2005 | Brazil | 2005 | EU582479 |  |
| BR305-2005 | Brazil | 2005 | EU582480 |  |
| BR307-2005 | Brazil | 2005 | EU582481 |  |
| BR319-2005 | Brazil | 2005 | EU582482 |  |
| SP118/2005 | Brazil | 2005 | EU625735 | (Souza *et al.,* unpublished) |
| SP661/2006 | Brazil | 2006 | EU625736 |  |
| JU667/2006 | Brazil | 2005 | EU625737 |  |
| SP691/2006 | Brazil | 2006 | EU625738 |  |
| SP725/2006 | Brazil | 2006 | EU625739 |  |
| SP773/2006 | Brazil | 2006 | EU625740 |  |
| SP779/2006 | Brazil | 2006 | EU625741 |  |
| SP876/2006 | Brazil | 2006 | EU625742 |  |
| SP900/2006 | Brazil | 2006 | EU625743 |  |
| SP902/2006 | Brazil | 2006 | EU625744 |  |
| SP912/2006 | Brazil | 2006 | EU625745 |  |
| SP914/2006 | Brazil | 2006 | EU625746 |  |
| SP915/2006 | Brazil | 2006 | EU625747 |  |
| SP918/2006 | Brazil | 2006 | EU625748 |  |
| SP928/2006 | Brazil | 2006 | EU625749 |  |
| SP929/2006 | Brazil | 2006 | EU625750 |  |
| SP931/2006 | Brazil | 2006 | EU625751 |  |
| SP955/2006 | Brazil | 2006 | EU625752 |  |
| SP956/2006 | Brazil | 2006 | EU625753 |  |
| SP958/2006 | Brazil | 2006 | EU625754 |  |
| SP974/2006 | Brazil | 2006 | EU625755 |  |
| SP987/2006 | Brazil | 2006 | EU625756 |  |
| SP999/2006 | Brazil | 2006 | EU625757 |  |
| SP1018/2006 | Brazil | 2006 | EU625758 |  |
| SP1016/2006 | Brazil | 2006 | EU625759 |  |
| SP1023/2006 | Brazil | 2006 | EU625760 |  |
| JU1042/2006 | Brazil | 2006 | EU625761 |  |
| SP1051/2006 | Brazil | 2006 | EU625762 |  |
| SP1052/2006 | Brazil | 2006 | EU625763 |  |
| SP1054/2006 | Brazil | 2006 | EU625764 |  |
| SP1057/2006 | Brazil | 2006 | EU625765 |  |
| SP1066/2006 | Brazil | 2006 | EU625766 |  |
| SP1067/2006 | Brazil | 2006 | EU625767 |  |
| SP1079/2006 | Brazil | 2006 | EU625768 |  |
| SP1104/2006 | Brazil | 2006 | EU625769 |  |
| JU1124/2006 | Brazil | 2006 | EU625770 |  |
| JU1125/2006 | Brazil | 2006 | EU625771 |  |
| JU1185/2006 | Brazil | 2006 | EU625772 |  |
| SP1189/2006 | Brazil | 2006 | EU625773 |  |
| SP1196/2006 | Brazil | 2006 | EU625774 |  |
| SP1218/2006 | Brazil | 2006 | EU625775 |  |
| JU1240/2006 | Brazil | 2006 | EU625776 |  |
| JU1324/2006 | Brazil | 2006 | EU625777 |  |
| SP1408/2006 | Brazil | 2006 | EU625778 |  |
| SP808/2006 | Brazil | 2006 | EU625779 |  |
| SP937/2006 | Brazil | 2006 | EU625780 |  |
| SP941/2006 | Brazil | 2006 | EU625781 |  |
| SP948/2006 | Brazil | 2006 | EU625782 |  |
| SP954/2006 | Brazil | 2006 | EU625783 |  |
| SP962/2006 | Brazil | 2006 | EU625784 |  |
| SP969/2006 | Brazil | 2006 | EU625785 |  |
| SP972/2006 | Brazil | 2006 | EU625786 |  |
| SP1058/2006 | Brazil | 2006 | EU625787 |  |
| SP1064/2006 | Brazil | 2006 | EU625788 |  |
| SP1086/2006 | Brazil | 2006 | EU625789 |  |
| JU1108/2006 | Brazil | 2006 | EU625790 |  |
| JU1159/2006 | Brazil | 2006 | EU625791 |  |
| JU1351/2006 | Brazil | 2006 | EU625792 |  |
| SP1746/2007 | Brazil | 2007 | EU625793 |  |
| SP1693/2007 | Brazil | 2007 | EU625794 |  |
| SP1759/2007 | Brazil | 2007 | EU625795 |  |
| SP1763/2007 | Brazil | 2007 | EU625796 |  |
| SP1787/2007 | Brazil | 2007 | EU625797 |  |
| SP1800/2007 | Brazil | 2007 | EU625798 |  |
| RP040/05 | Brazil | 2005 | EU635852 | (Proenca-Modena *et al*., unpublished) |
| RP050/05 | Brazil | 2005 | EU635853 |  |
| RP053/05 | Brazil | 2005 | EU635854 |  |
| RP056/05 | Brazil | 2005 | EU635855 |  |
| RP061/05 | Brazil | 2005 | EU635856 |  |
| RP066/05 | Brazil | 2005 | EU635857 |  |
| RP078/05 | Brazil | 2005 | EU635858 |  |
| RP082/05 | Brazil | 2005 | EU635859 |  |
| RP103/05 | Brazil | 2005 | EU635860 |  |
| RP108/05 | Brazil | 2005 | EU635861 |  |
| RP110/05 | Brazil | 2005 | EU635862 |  |
| RP125/05 | Brazil | 2005 | EU635863 |  |
| RP161/05 | Brazil | 2005 | EU635864 |  |
| RP193/05 | Brazil | 2005 | EU635865 |  |
| RP023/06 | Brazil | 2006 | EU635866 |  |
| RP033/06 | Brazil | 2006 | EU635867 |  |
| RP043/06 | Brazil | 2006 | EU635868 |  |
| RP045/06 | Brazil | 2006 | EU635869 |  |
| RP049/06 | Brazil | 2006 | EU635870 |  |
| RP062/06 | Brazil | 2006 | EU635871 |  |
| RP189/06 | Brazil | 2006 | EU635872 |  |
| BR/A-18/04 | Brazil | 2004 | FJ804089 | [7] |
| BR/A-27/04 | Brazil | 2004 | FJ804090 |  |
| MAD/2258/98 | Spain | 1998-1999 | GQ150687 | [8] |
| MAD/2259/98 | Spain | 1998-1999 | GQ150688 |  |
| MAD/2263/98 | Spain | 1998-1999 | GQ150689 |  |
| MAD/3553/01 | Spain | 2001-2002 | GQ150690 |  |
| MAD/3743/02 | Spain | 2002-2003 | GQ150691 |  |
| MAD/3765/02 | Spain | 2002-2003 | GQ150692 |  |
| MAD/4033/03 | Spain | 2003-2004 | GQ150693 |  |
| MAD/4073/03 | Spain | 2003-2004 | GQ150694 |  |
| MAD/4055/03 | Spain | 2003-2004 | GQ150695 |  |
| MAD/4139/03 | Spain | 2003-2004 | GQ150696 |  |
| MAD/4141/03 | Spain | 2003-2004 | GQ150697 |  |
| MAD/4163/03 | Spain | 2003-2004 | GQ150698 |  |
| MAD/4164/03 | Spain | 2003-2004 | GQ150699 |  |
| MAD/4165/03 | Spain | 2003-2004 | GQ150700 |  |
| MAD/4169/03 | Spain | 2003-2004 | GQ150701 |  |
| MAD/4515/04 | Spain | 2003-2004 | GQ150702 |  |
| MAD/4526/04 | Spain | 2003-2004 | GQ150703 |  |
| MAD/4527/04 | Spain | 2003-2004 | GQ150704 |  |
| MAD/4533/04 | Spain | 2003-2004 | GQ150705 |  |
| MAD/4535/04 | Spain | 2003-2004 | GQ150706 |  |
| MAD/4540/04 | Spain | 2003-2004 | GQ150707 |  |
| MAD/4546/04 | Spain | 2003-2004 | GQ150708 |  |
| MAD/4550/04 | Spain | 2003-2004 | GQ150709 |  |
| MAD/4557/04 | Spain | 2003-2004 | GQ150710 |  |
| MAD/4570/04 | Spain | 2003-2004 | GQ150711 |  |
| MAD/4573/04 | Spain | 2003-2004 | GQ150712 |  |
| MAD/4577/04 | Spain | 2003-2004 | GQ150713 |  |
| MAD/4591/04 | Spain | 2003-2004 | GQ150714 |  |
| MAD/4643/04 | Spain | 2003-2004 | GQ150715 |  |
| MAD/4668/04 | Spain | 2003-2004 | GQ150716 |  |
| MAD/4938/05 | Spain | 2005-2006 | GQ150717 |  |
| MAD/4950/05 | Spain | 2005-2006 | GQ150718 |  |
| MAD/4953/05 | Spain | 2005-2006 | GQ150719 |  |
| MAD/4956/05 | Spain | 2005-2006 | GQ150720 |  |
| MAD/4957/05 | Spain | 2005-2006 | GQ150721 |  |
| MAD/4993/05 | Spain | 2005-2006 | GQ150722 |  |
| MAD/5015/05 | Spain | 2005-2006 | GQ150723 |  |
| MAD/5058/05 | Spain | 2005-2006 | GQ150724 |  |
| MAD/5064/05 | Spain | 2005-2006 | GQ150725 |  |
| MAD/5097/05 | Spain | 2005-2006 | GQ150726 |  |
| MAD/5098/05 | Spain | 2005-2006 | GQ150727 |  |
| MAD/5144/05 | Spain | 2005-2006 | GQ150728 |  |
| MAD/5662/06 | Spain | 2006-2007 | GQ150729 |  |
| MAD/5670/06 | Spain | 2006-2007 | GQ150730 |  |
| MAD/5683/06 | Spain | 2006-2007 | GQ150731 |  |
| MAD/5688/06 | Spain | 2006-2007 | GQ150732 |  |
| MAD/5732/06 | Spain | 2006-2007 | GQ150733 |  |
| MAD/5745/06 | Spain | 2006-2007 | GQ150734 |  |
| MAD/5751/06 | Spain | 2006-2007 | GQ150735 |  |
| MAD/5755/06 | Spain | 2006-2007 | GQ150736 |  |
| MAD/6194/07 | Spain | 2007-2008 | GQ150737 |  |
| MAD/6198/07 | Spain | 2007-2008 | GQ150738 |  |
| MAD/6212/07 | Spain | 2007-2008 | GQ150739 |  |
| MAD/6214/07 | Spain | 2007-2008 | GQ150740 |  |
| MAD/6221/07 | Spain | 2007-2008 | GQ150741 |  |
| MAD/6222/07 | Spain | 2007-2008 | GQ150742 |  |
| MAD/6312/07 | Spain | 2007-2008 | GQ150743 |  |
| NG-068-05 | Japan | 2005 | HM459864 | [9] |
| NG-082-07 | Japan | 2007 | HM459868 |  |
| NG-228-06 | Japan | 2006 | HM459871 |  |
| NG-013-07 | Japan | 2007 | HM459873 |  |
| NG-149-06 | Japan | 2006 | HM459877 |  |
| NG-015-09 | Japan | 2009 | HM459880 |  |
| NG-084-07 | Japan | 2007 | HM459883 |  |
| NG-047-07 | Japan | 2007 | HM459887 |  |
| NG-065-07 | Japan | 2007 | HM459891 |  |
| SA481582OP06 | South Africa | 2006 | HQ7117811 | [10] |
| SA459004OP06 | South Africa | 2006 | HQ7117831 |  |
| SA1904180OP09 | South Africa | 2009 | HQ7117841 |  |
| SA622525OP06 | South Africa | 2006 | HQ7117851 |  |
| SA1906931OP09 | South Africa | 2009 | HQ7117861 |  |
| SA1898386H09 | South Africa | 2009 | HQ7117871 |  |
| SA475995H06 | South Africa | 2006 | HQ7117881 |  |
| SA957529OP07 | South Africa | 2007 | HQ7117891 |  |
| SA430310OP06 | South Africa | 2006 | HQ7117901 |  |
| SA432203OP06 | South Africa | 2006 | HQ7117911 |  |
| SA451653OP06 | South Africa | 2006 | HQ7117921 |  |
| SA523250OP06 | South Africa | 2006 | HQ7117931 |  |
| SA430319H06 | South Africa | 2006 | HQ7117941 |  |
| SA451774H06 | South Africa | 2006 | HQ7117951 |  |
| SA435745OP06 | South Africa | 2006 | HQ7117961 |  |
| SA490490H06 | South Africa | 2006 | HQ7117971 |  |
| SA983753OP07B | South Africa | 2007 | HQ7117981 |  |
| SA1138929H07 | South Africa | 2007 | HQ7117991 |  |
| SA472200OP06 | South Africa | 2006 | HQ7118001 |  |
| SA539486H06 | South Africa | 2006 | HQ7118011 |  |
| SA433941OP06 | South Africa | 2006 | HQ7118021 |  |
| SA555134H06 | South Africa | 2006 | HQ7118031 |  |
| SA466313H06B | South Africa | 2006 | HQ7118041 |  |
| SA539456H06B | South Africa | 2006 | HQ7118051 |  |
| SA954715OP07B | South Africa | 2007 | HQ7118061 |  |
| SA921110H07 | South Africa | 2007 | HQ7118071 |  |
| SA404376OP06 | South Africa | 2006 | HQ7118081 |  |
| SA1961261H09 | South Africa | 2009 | HQ7118091 |  |
| SA1903163H09 | South Africa | 2009 | HQ7118101 |  |
| SA1897566H09 | South Africa | 2009 | HQ7118111 |  |
| SA1946046H09 | South Africa | 2009 | HQ7118121 |  |
| SA1914360H09 | South Africa | 2009 | HQ7118131 |  |
| SA483388OP06 | South Africa | 2006 | HQ7118141 |  |
| SA574601H06 | South Africa | 2006 | HQ7118151 |  |
| SA1507096OP08 | South Africa | 2008 | HQ7118161 |  |
| SA1913535H09 | South Africa | 2009 | HQ7118171 |  |
| SA1559601OP08 | South Africa | 2008 | HQ7118181 |  |
| SA1856310OP09 | South Africa | 2009 | HQ7118191 |  |
| SA581105H06 | South Africa | 2006 | HQ7118201 |  |
| SA1952519H09 | South Africa | 2009 | HQ7118211 |  |
| SA1903864H09 | South Africa | 2009 | HQ7118221 |  |
| SA1966323H09 | South Africa | 2009 | HQ7118231 |  |
| SA1945943H09 | South Africa | 2009 | HQ7118241 |  |
| SA1856312OP09 | South Africa | 2009 | HQ7118251 |  |
| SA1954125H09 | South Africa | 2009 | HQ7118261 |  |
| SA1901413H09 | South Africa | 2009 | HQ711827 |  |
| SA1023607OP07 | South Africa | 2007 | HQ711828 |  |
| SA1510114OP08 | South Africa | 2008 | HQ711829 |  |
| SA1911347OP09 | South Africa | 2009 | HQ711830 |  |
| SA1037074H07 | South Africa | 2007 | HQ711831 |  |
| SA1864925OP09 | South Africa | 2009 | HQ711832 |  |
| SA1960816H09 | South Africa | 2009 | HQ711833 |  |
| SA466303OP06 | South Africa | 2006 | HQ711834 |  |
| SA530589OP06B | South Africa | 2006 | HQ711835 |  |
| SA1918530OP09 | South Africa | 2009 | HQ711836 |  |
| SA453610H06 | South Africa | 2009 | HQ711837 |  |
| SA512554H06 | South Africa | 2006 | HQ711838 |  |
| SA417463H06 | South Africa | 2006 | HQ711839 |  |
| SA/581105Pt/06 | South Africa | 2006 | JF704219 | [11] |
| SA/466303Pt/06 | South Africa | 2006 | JF704220 |  |
| SA486540Pt06 | South Africa | 2006 | JF704221 |  |
| SA/539456Pt/06 | South Africa | 2006 | JF704222 |  |
| SA/555134Pt/06 | South Africa | 2006 | JF704223 |  |
| SA/957529Pt/07 | South Africa | 2007 | JF704224 |  |
| SA/983753Pt/07 | South Africa | 2007 | JF704224 |  |
| SA/1023607Pt/07 | South Africa | 2007 | JF704226 |  |
| SA/1138929Pt/08 | South Africa | 2008 | JF704227 |  |
| SA/1510114Pt/08 | South Africa | 2008 | JF704228 |  |
| SA/1904180Pt/09 | South Africa | 2009 | JF704229 |  |
| SA/1906931Pt/09 | South Africa | 2009 | JF704230 |  |
| SA/1918530Pt/09 | South Africa | 2009 | JF704231 |  |
| SA/1917319Pt/09 | South Africa | 2009 | JF704232 |  |
| SA/1954125Pt/09 | South Africa | 2009 | JF704233 |  |
| SA/1913535Pt/09 | South Africa | 2009 | JF704234 |  |
| B/WI/629-5B/06-07 | USA | 2006-2007 | JN032115 | [12] |
| B/WI/629-12/06-07 | USA | 2006-2007 | JN032116 |  |
| B/WI/629-15/06-07 | USA | 2006-2007 | JN0321171 |  |
| B/WI/629-DC1/08-09 | USA | 2008-2009 | JN032119 |  |
| Cam2005-0038 | Cambodia | 2005 | JN119953 | [13] |
| Cam2005-0122 | Cambodia | 2005 | JN119954 |  |
| Cam2005-0122 | Cambodia | 2005 | JN119954 |  |
| Cam2005-0122 | Cambodia | 2005 | JN119954 |  |
| Cam2005-7292 | Cambodia | 2005 | JN119955 |  |
| Cam2005-7293 | Cambodia | 2005 | JN119956 |  |
| Cam2005-7296 | Cambodia | 2005 | JN119957 |  |
| Cam2005-7297 | Cambodia | 2005 | JN119958 |  |
| Cam2005-7297 | Cambodia | 2005 | JN119958 |  |
| Cam2005-7300 | Cambodia | 2005 | JN119959 |  |
| Cam2007-6084 | Cambodia | 2007 | JN119960 |  |
| Cam2008-1045 | Cambodia | 2008 | JN119961 |  |
| Cam2008-1102 | Cambodia | 2008 | JN119962 |  |
| Cam2008-1147 | Cambodia | 2008 | JN119963 |  |
| Cam2008-3125 | Cambodia | 2008 | JN119964 |  |
| Cam2008-3246 | Cambodia | 2008 | JN119965 |  |
| Cam2008-3393 | Cambodia | 2008 | JN119966 |  |
| Cam2008-5284 | Cambodia | 2008 | JN119967 |  |
| Cam2008-5285 | Cambodia | 2008 | JN119968 |  |
| Cam2008-7298 | Cambodia | 2008 | JN119970 |  |
| Cam2008-7298 | Cambodia | 2008 | JN119970 |  |
| Cam2008-8175 | Cambodia | 2008 | JN119971 |  |
| Cam2008-9297 | Cambodia | 2008 | JN119972 |  |
| Cam2009-0049 | Cambodia | 2009 | JN119973 |  |
| Cam2009-0078 | Cambodia | 2009 | JN119974 |  |
| Cam2009-0124 | Cambodia | 2009 | JN119975 |  |
| Cam2009-0351 | Cambodia | 2009 | JN119976 |  |
| Cam2009-0370 | Cambodia | 2009 | JN119977 |  |
| Cam2009-0396 | Cambodia | 2009 | JN119978 |  |
| Cam2009-0396 | Cambodia | 2009 | JN119978 |  |
| Cam2009-1019 | Cambodia | 2009 | JN119980 |  |
| Cam2009-1027 | Cambodia | 2009 | JN119981 |  |
| Cam2009-1240 | Cambodia | 2009 | JN119982 |  |
| Cam2009-2106 | Cambodia | 2009 | JN119983 |  |
| Cam2009-2120 | Cambodia | 2009 | JN119984 |  |
| Cam2009-2125 | Cambodia | 2009 | JN119985 |  |
| Cam2009-2141 | Cambodia | 2009 | JN119986 |  |
| Cam2009-2153 | Cambodia | 2009 | JN119988 |  |
| Cam2009-3035 | Cambodia | 2009 | JN119990 |  |
| Cam2009-5023 | Cambodia | 2009 | JN119991 |  |
| Cam2009-5034 | Cambodia | 2009 | JN119992 |  |
| Cam2009-5090 | Cambodia | 2009 | JN119993 |  |
| Cam2009-5090 | Cambodia | 2009 | JN119993 |  |
| Cam2009-5091 | Cambodia | 2009 | JN119994 |  |
| Cam2009-5148 | Cambodia | 2009 | JN119995 |  |
| Cam2009-5149 | Cambodia | 2009 | JN119996 |  |
| Cam2009-5157 | Cambodia | 2009 | JN119997 |  |
| Cam2009-5169 | Cambodia | 2009 | JN119998 |  |
| Cam2009-6080 | Cambodia | 2009 | JN120000 |  |
| Cam2009-7352 | Cambodia | 2009 | JN120004 |  |
| Cam2009-8162 | Cambodia | 2009 | JN120006 |  |
| Cam2009-8168 | Cambodia | 2009 | JN120008 |  |
| Cam2009-8186 | Cambodia | 2009 | JN120009 |  |
| Cam2009-8197 | Cambodia | 2009 | JN120010 |  |
| Cam2009-8233 | Cambodia | 2009 | JN120011 |  |
| Cam2009-8233 | Cambodia | 2009 | JN120011 |  |
| Cam2009-9062 | Cambodia | 2009 | JN120012 |  |
| Cam2009-9088 | Cambodia | 2009 | JN120013 |  |
| Cam2009-9165 | Cambodia | 2009 | JN120014 |  |
| Cam2009-9261 | Cambodia | 2009 | JN120015 |  |
| Cam2009-9265 | Cambodia | 2009 | JN120016 |  |
| Cam2009-9266 | Cambodia | 2009 | JN120017 |  |
| Cam2009-9269 | Cambodia | 2009 | JN120018 |  |
| Cam2009-9329 | Cambodia | 2009 | JN120019 |  |
| V08-2230920 | Hong Kong | 2008 | JN968343 | (Mak *et al*., unpublished) |
| V10-2224020 | Hong Kong | 2010 | JN968345 |  |
| V09-2826625 | Hong Kong | 2009 | JN968346 |  |
| V05-2216103 | Hong Kong | 2005 | JN968347 |  |
| V06-2252330 | Hong Kong | 2006 | JN968348 |  |
| V07-2272086 | Hong Kong | 2007 | JN968349 |  |
| V08-2255950 | Hong Kong | 2008 | JN968350 |  |
| V10-2210252 | Hong Kong | 2010 | JN968351 |  |
| V09-2219184 | Hong Kong | 2009 | JN968352 |  |
| V10-2230104 | Hong Kong | 2010 | JN968353 |  |
| V09-2272107 | Hong Kong | 2009 | JN968354 |  |
| V10-2259743 | Hong Kong | 2010 | JN968355 |  |
| V10-2241704 | Hong Kong | 2010 | JN968356 |  |
| V06-2207926 | Hong Kong | 2006 | JN968357 |  |
| V10-2204103 | Hong Kong | 2010 | JN968358 |  |
| V09-2228367 | Hong Kong | 2009 | JN968359 |  |
| V04-2204047 | Hong Kong | 2004 | JN968360 |  |
| V05-2238305 | Hong Kong | 2005 | JN968361 |  |
| V09-2851524 | Hong Kong | 2009 | JN968362 |  |
| V10-2223429 | Hong Kong | 2010 | JN968363 |  |
| V05-2227144 | Hong Kong | 2005 | JN968364 |  |
| V05-2257993 | Hong Kong | 2005 | JN968365 |  |
| V04-2247769 | Hong Kong | 2004 | JN968366 |  |
| BE/1346/10 | Belgium | 2010 |  | This study |
| BE/1649/10 | Belgium | 2010 |  |  |
| BE/225/10 | Belgium | 2010 |  |  |
| BE/271/07 | Belgium | 2007 |  |  |
| BE/324/07 | Belgium | 2007 |  |  |
| BE/3301859/09 | Belgium | 2009 |  |  |
| BE/333/11 | Belgium | 2011 |  |  |
| BE/3354412/10 | Belgium | 2010 |  |  |
| BE/3374058/10 | Belgium | 2010 |  |  |
| BE/404/10 | Belgium | 2010 |  |  |
| BE/46/03 | Belgium | 2003 |  |  |
| BE/46/08 | Belgium | 2008 |  |  |
| BE/4948/08 | Belgium | 2008 |  |  |
| BE/5149/08 | Belgium | 2008 |  |  |
| BE/5150/08 | Belgium | 2008 |  |  |
| BE/5159/08 | Belgium | 2008 |  |  |
| BE/5213/08 | Belgium | 2008 |  |  |
| BE/5312/08 | Belgium | 2008 |  |  |
| BE/5351/08 | Belgium | 2008 |  |  |
| BE/5354/07 | Belgium | 2007 |  |  |
| BE/539/11 | Belgium | 2011 |  |  |
| BE/5394/08 | Belgium | 2008 |  |  |
| BE/5423/08 | Belgium | 2008 |  |  |
| BE/5437/08 | Belgium | 2008 |  |  |
| BE/5449/07 | Belgium | 2007 |  |  |
| BE/5489/08 | Belgium | 2008 |  |  |
| BE/5491/08 | Belgium | 2008 |  |  |
| BE/5494/08 | Belgium | 2008 |  |  |
| BE/553/03 | Belgium | 2003 |  |  |
| BE/5533/08 | Belgium | 2008 |  |  |
| BE/5649/08 | Belgium | 2008 |  |  |
| BE/5691/08 | Belgium | 2008 |  |  |
| BE/5730/08 | Belgium | 2008 |  |  |
| BE/5823/08 | Belgium | 2008 |  |  |
| BE/5910/08 | Belgium | 2008 |  |  |
| BE/5923/06 | Belgium | 2006 |  |  |
| BE/5983/08 | Belgium | 2008 |  |  |
| BE/6007/07 | Belgium | 2007 |  |  |
| BE/6090/07 | Belgium | 2007 |  |  |
| BE/6156/07 | Belgium | 2007 |  |  |
| BE/6259/08 | Belgium | 2008 |  |  |
| BE/6316/08 | Belgium | 2008 |  |  |
| BE/6346/06 | Belgium | 2006 |  |  |
| BE/6504/08 | Belgium | 2008 |  |  |
| BE/6548/10 | Belgium | 2010 |  |  |
| BE/6567/06 | Belgium | 2006 |  |  |
| BE/6569/10 | Belgium | 2010 |  |  |
| BE/6649/06 | Belgium | 2006 |  |  |
| BE/6668/06 | Belgium | 2006 |  |  |
| BE/6706/06 | Belgium | 2006 |  |  |
| BE/6726/07 | Belgium | 2007 |  |  |
| BE/6734/09 | Belgium | 2009 |  |  |
| BE/6737/06 | Belgium | 2006 |  |  |
| BE/6738/06 | Belgium | 2006 |  |  |
| BE/6741/06 | Belgium | 2006 |  |  |
| BE/6797/07 | Belgium | 2007 |  |  |
| BE/6857/06 | Belgium | 2006 |  |  |
| BE/6886/06 | Belgium | 2006 |  |  |
| BE/6939/06 | Belgium | 2006 |  |  |
| BE/6944/06 | Belgium | 2006 |  |  |
| BE/6953/07 | Belgium | 2007 |  |  |
| BE/7102/06 | Belgium | 2006 |  |  |
| BE/7163/06 | Belgium | 2006 |  |  |
| BE/7176/07 | Belgium | 2007 |  |  |
| BE/7254/06 | Belgium | 2006 |  |  |
| BE/7269/06 | Belgium | 2006 |  |  |
| BE/7407/06 | Belgium | 2006 |  |  |
| BE/7539/06 | Belgium | 2006 |  |  |
| BE/7848/10 | Belgium | 2010 |  |  |
| BE/7852/10 | Belgium | 2010 |  |  |
| BE/8/11 | Belgium | 2011 |  |  |
| BE/8777/09 | Belgium | 2009 |  |  |
| BE/8844/09 | Belgium | 2009 |  |  |
| BE/8845/09 | Belgium | 2009 |  |  |
| BE/8933/09 | Belgium | 2009 |  |  |
| BE/9004/09 | Belgium | 2009 |  |  |
| BE/9109/09 | Belgium | 2009 |  |  |
| BE/9125/09 | Belgium | 2009 |  |  |
| BE/9141/09 | Belgium | 2009 |  |  |
| BE/9207/09 | Belgium | 2009 |  |  |
| BE/9220/09 | Belgium | 2009 |  |  |
| BE/9295/09 | Belgium | 2009 |  |  |
| BE/9364/09 | Belgium | 2009 |  |  |
| BE/936751/08 | Belgium | 2008 |  |  |
| BE/938609/08 | Belgium | 2008 |  |  |
| BE/939024/08 | Belgium | 2008 |  |  |
| BE/939189/08 | Belgium | 2008 |  |  |
| BE/939641/08 | Belgium | 2008 |  |  |
| BE/94/07 | Belgium | 2007 |  |  |
| BE/940667/08 | Belgium | 2008 |  |  |
| BE/940668/08 | Belgium | 2008 |  |  |
| BE/944484/08 | Belgium | 2008 |  |  |
| BE/944527/08 | Belgium | 2008 |  |  |
| BE/950755/08 | Belgium | 2008 |  |  |
| BE/954893/08 | Belgium | 2008 |  |  |
| BE/956911/08 | Belgium | 2008 |  |  |
| BE/962837/08 | Belgium | 2008 |  |  |

1. Nagai K, Kamasaki H, Kuroiwa Y, Okita L, Tsutsumi H (2004) Nosocomial outbreak of respiratory syncytial virus subgroup B variants with the 60 nucleotides-duplicated G protein gene. J Med Virol 74: 161-165.

2. Trento A, Viegas M, Galiano M, Videla C, Carballal G, et al. (2006) Natural history of human respiratory syncytial virus inferred from phylogenetic analysis of the attachment (G) glycoprotein with a 60-nucleotide duplication. Journal of Virology 80: 975-984.

3. Zlateva KT, Lemey P, Moes E, Vandamme AM, Van Ranst M (2005) Genetic variability and molecular evolution of the human respiratory syncytial virus subgroup B attachment G protein. J Virol 79: 9157-9167.

4. Matheson JW, Rich FJ, Cohet C, Grimwood K, Huang QS, et al. (2006) Distinct patterns of evolution between respiratory syncytial virus subgroups A and B from New Zealand isolates collected over thirty-seven years. J Med Virol 78: 1354-1364.

5. Parveen S, Sullender WM, Fowler K, Lefkowitz EJ, Kapoor SK, et al. (2006) Genetic variability in the G protein gene of group A and B respiratory syncytial viruses from India. Journal of Clinical Microbiology 44: 3055-3064.

6. Visser A, Delport S, Venter M (2008) Molecular epidemiological analysis of a nosocomial outbreak of respiratory syncytial virus associated pneumonia in a kangaroo mother care unit in South Africa. J Med Virol 80: 724-732.

7. da Silva LH, Spilki FR, Riccetto AG, de Almeida RS, Baracat EC, et al. (2008) Genetic variability in the G protein gene of human respiratory syncytial virus isolated from the Campinas metropolitan region, Brazil. J Med Virol 80: 1653-1660.

8. Trento A, Casas I, Calderon A, Garcia-Garcia ML, Calvo C, et al. (2010) Ten years of global evolution of the human respiratory syncytial virus BA genotype with a 60-nucleotide duplication in the G protein gene. J Virol 84: 7500-7512.

9. Dapat IC, Shobugawa Y, Sano Y, Saito R, Sasaki A, et al. (2010) New genotypes within respiratory syncytial virus group B genotype BA in Niigata, Japan. J Clin Microbiol 48: 3423-3427.

10. van Niekerk S, Venter M (2011) Replacement of previously circulating Respiratory Syncytial Virus (RSV) subtype B strains with the BA genotype in South Africa. J Virol.

11. Venter M, van Niekerk S, Rakgantso A, Bent N (2011) Identification of deletion mutant respiratory syncytial virus strains lacking most of the G protein in immunocompromised children with pneumonia in South Africa. Journal of Virology 85: 8453-8457.

12. Rebuffo-Scheer C, Bose M, He J, Khaja S, Ulatowski M, et al. (2011) Whole genome sequencing and evolutionary analysis of human respiratory syncytial virus A and B from Milwaukee, WI 1998-2010. PLoS One 6: e25468.

13. Arnott A, Vong S, Mardy S, Chu S, Naughtin M, et al. (2011) A study of the genetic variability of human respiratory syncytial virus (HRSV) in Cambodia reveals the existence of a new HRSV group B genotype. Journal of Clinical Microbiology 49: 3504-3513.
